# The Combination of Exercise and Konjac Glucomannan More Effectively Prevents Antibiotics-Induced Dysbiosis in Mice Compared with Singular Intervention

**DOI:** 10.3390/nu16172942

**Published:** 2024-09-02

**Authors:** Minghan Wang, Yonglin Chen, Ang-Xin Song, Xiquan Weng, Yan Meng, Jieru Lin, Yu-Heng Mao

**Affiliations:** 1 School of Exercise and Health, Guangzhou Sport University, Guangzhou 510500, China; 2 Key Laboratory of Plant Resource Conservation and Germplasm Innovation in Mountainous Region (Ministry of Education), School of Liquor and Food Engineering, Guizhou University, Guiyang 550025, China; 3 Guangdong Key Laboratory of Human Sports Performance Science, Guangzhou Sport University, Guangzhou 510500, China

**Keywords:** glucomannan dietary fiber, sports, gut microbiome, metagenomics, SCFA

## Abstract

Our previous studies have demonstrated that konjac glucomannan (KGM) can prevent dysbiosis induced by antibiotics. While exercise may also impact the gut microbiome, there are limited studies reporting its protective effect on antibiotic-induced dysbiosis. Therefore, this study investigated the preventive and regulatory effects of a combination of 6-week exercise and KGM intervention on antibiotic-induced dysbiosis in C57BL/6J mice compared with a single intervention. The results showed that combined exercise and KGM intervention could restore the changes in the relative abundance of *Bacteroides* (3.73% with CTL versus 14.23% with ATBX versus 4.46% with EK) and *Prevotellaceae_Prevotella* (0.33% with CTL versus 0.00% with ATBX versus 0.30% with EK) induced by antibiotics (*p* < 0.05), and minimized the Bray–Curtis distance induced by antibiotics (0.55 with CTL versus 0.81 with ATBX versus 0.80 with EXC versus 0.83 with KGM versus 0.75 with EK). Compared with the combined intervention, exercise intervention also produced a certain level of recovery effects; the relative abundance of Rikenellaceae (1.96% with CTL versus 0.09% with ATBX versus 0.49% with EXC) was restored, while KGM supplementation showed the best preventive effect. In addition, the combination of exercise and KGM significantly enriched microbial purine metabolic pathways (*p* < 0.05). These findings indicate that combining exercise with KGM could be a promising approach to reducing the side effects of antibiotics on the gut microbiome.

## 1. Introduction

Antibiotics are highly effective in reducing bacterial infections and restoring good health and are widely utilized in clinical practices; in a population experiment, participants who received continuous antibiotic prophylaxis for 24 months were 14.4% less likely to develop a first urinary tract infection than untreated participants [1]. However, the inappropriate use of antibiotics can also lead to other health issues [2], such as triggering allergic reactions and toxic responses [3], as well as negatively impacting the gut microbiome, leading to gut microbiome dysbiosis [4] and causing gastrointestinal symptoms and other systemic diseases [5].

Microecological preparations mainly include probiotics, prebiotics, and synbiotics, which can regulate the intestinal microecological balance, improve the health level of the host, and promote the health state of the physiological live bacteria products and the metabolic products of these bacteria and promote the growth and reproduction of these physiological bacteria; they are considered an effective treatment for addressing dysbiosis resulting from antibiotic use [6,7,8]. According to our previous research, konjac glucomannan (KGM), a high molecular weight polysaccharide derived from konjac isolated from the tuber of *Amorphophallus konjac C. Koch.*, has demonstrated its ability to prevent and regulate antibiotic-induced dysbiosis in the gut microbiome [9].

Exercise is an effective and personalized strategy to promote physical health and prevent disease. It can regulate metabolic function, boost the immune system, impact inflammation status, and ultimately improve overall quality of life [10]. Emerging evidence has demonstrated that exercise can influence the composition and distribution of the gut microbiome [11]. This includes regulating the richness and diversity of microorganisms, balancing intestinal microbiota through increased colonization of beneficial bacteria, and improving host immune function and metabolic capacity [12]. In animal models, the movement distance of the animals was negatively correlated with Bacteroidetes, and the proportion of harmful bacteria in the exercise mice was also significantly reduced [12]. Despite these findings, there is currently no research on whether exercise can prevent or regulate antibiotic-induced dysbiosis in the gut microbiota.

Therefore, this study aimed to investigate the impact of a singular aerobic exercise intervention and its combination with KGM on dysbiosis induced by antibiotics. The combination of nutritional and sports interventions is widely recognized as the most effective strategy for managing many chronic diseases. The combination of diet control and exercise is definitely more effective than a singular intervention and is well-documented in various chronic disease treatment, such as obesity [13], and based on the positive effects of exercise and KGM on gut microbiome [14,15], we hypothesize that the combination of exercise and KGM intervention is more effective in preventing dysbiosis and regulating the gut microbiome. This study holds significance in searching for potential solutions for better-managing dysbiosis.

## 2. Materials and Methods

### 2.1. Chemicals

Based on our previous study [9], the native KGM with the highest molecular weight was used in this study. A KGM with a purity of >95% was purchased from Johnson & Johnson (Ezhou, China). The KGM used in the study had a molecular weight of 1.82 × 10^7^ Da, a specific viscosity of 9.48 dL/g, and a galactose/glucose ratio of 1.65:1. The six short-chain fatty acids standards, including acetic acid, propionic acid, *n*-butyric acid, *iso*-butyric acid, *n*-valeric acid, and *iso*-valeric acid, were purchased from Aradin (Shanghai, China). A DNA extraction kit was purchased from Tiangen Biotechnology Co., Ltd. (Beijing, China). All the chemicals used in this study are listed in Table S1, and the detailed information is from the NCBI PubChem Compound Database and the supplier sources.

### 2.2. Animal Experiment Design

Thirty male C57BL/6J mice (7-week-old) were purchased from Zhuhai BesTest Bio-Tech Co., Ltd. (Zhuhai, Guangdong, China) and randomly assigned to 5 groups. They were pre-adapted for 1 week prior to the experiment. Throughout the study, the mice were provided with standard feed (AIN-93G purified feed, detailed formula listed in Table S2) and distilled water. To eliminate potential confounding effects of female hormones on experimental data, only male mice were used as subjects in this study. The mice were housed in specific pathogen-free (SPF) facilities at Guangzhou Sport University Animal Center under standard conditions (22–24 °C, 50 ± 5% humidity), following a 12 h light/dark cycle. All experimental procedures adhered to the NRC Guidelines for the Care and Use of Laboratory Animals (2011). The animal experiment was reviewed and approved by the Animal Experimental Ethics Inspection of Guangzhou Sport University (Permit No. 2022DWLL-24, 19 August 2022).

**Figure 1:**
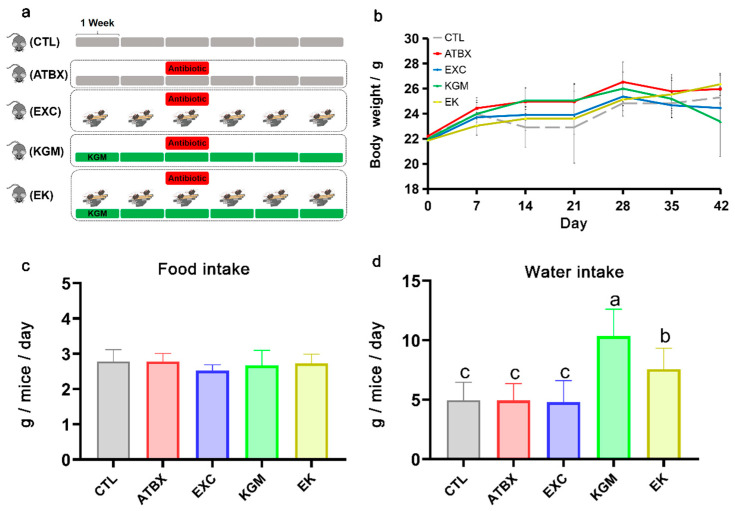
(**a**) The animal experiment diagram, (**b**) body weight, (**c**) food intake, (**d**) water intake in different groups for 42 days. Average of six samples. Error bars represent standard deviation at N = 6. Different letters indicate the significant difference among different groups for the same index, ANOVA with Bonferroni or Tamhane T2 post-hoc test, *p* < 0.05. CTL: control; ATBX: antibiotic; EXC: exercise; KGM: the native KGM; EK: combination of exercise and KGM.

In order to comply with the prevention time of KGM and ensure that the exercise duration meets the minimum standard for long-term exercise intervention, we implemented a 6-week experimental period [16]. Further details regarding the intervention time were discussed in the Discussion section. As depicted in Figure 1a, during the 6-week experiment, mice in the control group (CTL), antibiotic group (ATBX), and exercise group (EXC) were provided with distilled water as their drinking water. The KGM group (KGM) and combined intervention group (EK) received KGM at a concentration of 2.5 g/L in their drinking water. During week 3, except for the CTL group, the other four groups received a combination of antibiotics (based on our previous study [9], the antibiotic formulation is ampicillin, streptomycin, and clindamycin at a ratio of 1 mg/mL each) added to their drinking water or KGM solution. The mice were free to access a drinking source and food. The water and food were replaced every two to three days, while the consumption of food and water was recorded and calculated weekly based. Based on the recorded data of provided and leftover weights of food and water, the total and daily average food intake and water intake of each mouse can be calculated throughout the experimental period. Mice underwent exercise training on the ZH-PT/5S treadmill (Zhenghua Bio-Instrument Facility, Huaibei, Anhui, China), with the EXC and EK groups undergoing a 6-week training regimen. The exercise protocol was designed based on a previously reported study, with slight modifications [17].

As depicted in Table 1, the exercise program adopts progressive load exercise and determines the treadmill speed in the first week at 60% of the maximum running speed. Due to the reported impact of antibiotic intervention on the exercise ability of mice [18], the exercise load was kept unchanged in week 3 (13 m/min, 40 min), as it was in week 2. For the same reason, the speed increased in week 4 (14 m/min, 40 min) and remained at that level until week 6.

Fecal samples were collected from all groups on day 0, 14, 21, and 42 and stored at −80 °C in sterile tubes for subsequent analysis. We strictly adhered to the 3R principles in our experiments. In cases where the body condition of mice was poor, stool collection and exercise intervention were suspended until they returned to a normal state. Following the endurance test, the mice were given a day’s rest and subsequently anesthetized with pentobarbital sodium. No incidents occurred at the end of the experiment. Blood samples were collected by cardiac puncture using a needle containing a solution of citric acid and glucose. The samples were immediately centrifuged at 3000 rpm at 4 °C for 15 min (Sorvall ST 8R small benchtop centrifuge, Thermo Fisher Scientific, Osterode am Harz, Germany), then plasma was collected and stored in a refrigerator at −80 °C for subsequent analysis.

### 2.3. Endurance Test

The mice were initially subjected to a speed of 10 m/min and underwent a 10 min running session, followed by incremental increases in treadmill speed every 2 min by 2 m/min until reaching exhaustion, enabling the assessment of their endurance performance [19]. According to our previous study [20], exhaustion was defined as the point at which the mice reached the electric grid at least five times within one minute or the inability of the animal to run on the treadmill for 10 s despite electrical prodding. Measures of mice exercise endurance included exhaustion time and running distance.

### 2.4. Histological Analysis

After rinsing the colon tissue with normal saline, it was subsequently fixed in a 4% paraformaldehyde solution at 4 °C for a duration of 24 h. Following fixation, the tissue was embedded in paraffin and subjected to hematoxylin and eosin (H and E) staining. A histological examination was performed using the Pannoramic 250 FLASH system (3DHISTECH Ltd., Budapest, Hungary), and an intestinal histological score was assigned (Table S3). The differences in intestinal pathological changes between different groups were analyzed utilizing CaseViewer Software 2.4.

### 2.5. Quantification of Short-Chain Fatty Acids in Plasma and Fecal Samples

Short chain fatty acids (SCFAs) in plasma and feces were determined using Shimadzu gas chromatograph (GC2010PLUS, Nishinokyo Kuwabara-cho, Nakagyo-ku, Kyoto 604-8511, Japan) based on previous studies with minor modifications [21]. The six SCFA standards used for identification and quantification include acetic acid, propionic acid, *n*-butyric acid, *iso*-butyric acid, *n*-valeric acid, and *iso*-valeric acid (Aladdin^®^, Shanghai, China). In the final calculation, *n*- and *iso*-butyric acid and *n*-and *iso*-valeric acid were combined to be butyric acid and valeric acid, respectively.

A volume of 150 mL of Milli-Q water was added in the sterilized tubes containing feces, followed by vortexing to complete homogenization. Then, the supernatant was collected after 2 rounds of centrifugation at 12,000 rpm, 4 °C for 15 min using a Sorvall ST 8R small benchtop centrifuge (Thermo Fisher Scientific, Osterode am Harz, Germany). The pH of the supernatant was adjusted to 2–3 using 1M HCl. Crotonic acid was used as the internal standard. The sample was injected after filtration with a 0.45 μm membrane.

Agilent 7890B gas chromatography was used to determine SCFAs. Shimadzu SH-Rtx-Wax capillary column (DB-FFAP 1233232, 30 × 0.32 mm, Agilent Technologies Inc., Santa Clara, CA, USA) was used for separation. The carrier gas was high-purity nitrogen, and the flow rate was 0.6 mL/min. The initial column temperature (100 °C) was maintained for 2 min, and the temperature was raised to 150 °C at a rate of 5 °C/min for 2 min and then heated to 200 °C at a rate of 20 °C/min for 1 min. The FID temperature was 240 °C.

### 2.6. DNA Extraction and 16S rRNA Sequencing

Genomic DNA extraction was performed using the Tiangen Fecal DNA Extraction Kit (Tiangen, Beijing, China) and was carried out according to the manufacturer’s instructions. The DNA concentration of microbial DNA was quantified using a NanoDrop 2000 (Thermo Fisher, Waltham, MA, USA). Using Wekemo Technology Co., Ltd. (Shenzhen, China), 16S rRNA gene sequencing was performed. The data were demultiplexed and adapter-trimmed using a DADA2 plugin in a Qiime2 software, resulting in amplicon sequence variants (ASVs). Then, species annotation was obtained by referring to a Greengenes database 13_8.

### 2.7. Metagenomic Sequencing and Data Analysis of Fecal Samples

The fecal samples were sent to Wekemo Technology Co., Ltd. (Shenzhen, Guangdong, China) for metagenomic sequencing and data analysis of the gut. DNA was extracted according to the method in previous studies [22]; DNA purity and concentration were determined by agarose gel electrophoresis. Metagenomic sequencing using the Illumina NovaSeq platform (San Diego, CA, USA) was conducted, employing insert sizes of 350 bp and paired-end reads of 150 bp for each sample. Elimination of substandard and indeterminate bases was performed on the raw reads. The key step for quality control is to first remove the joint sequence then scan the rest of the sequence; if the average quality score is less than 20 (99% accuracy), then cut the subsequent sequence and remove the final length of the sequence less than 50 bp. (Trimmomatic parameters: illuminclip: adapter path: 2:30:10, sliding window: 4:20, minlen: 50). The effective sequences were obtained by eliminating the reads that were aligned to both the rat’s genome reference and host DNA contamination, using Bowtie 2 with a parameter set as “very sensitive”. A Kraken2 (2018) program was utilized to analyze the diversity and composition of species. The DNA sequence abundance of each metagenomic sample was calculated using the Bayesian resampling abundance as a statistical method based on the Kraken2 results. According to the results of Bracken, the percentage of sequences from the kingdom to the species was obtained for each sample in the total sequence. Principal component analysis (PCA) showed overall differences in species composition between groups. The similarity of species composition was studied by cluster analysis.

Linear discriminant analysis (LDA) effect size (LEfSe) was used to identify the characteristic species with high abundance in various group. The thresholds of LDA > 2 and LDA > 4 were used to distinguish high-abundance species. Gene function analysis was conducted by employing the HUMAnN 2.0 software, which involved comparing DNA sequences, post-quality-control, and removal of host sequences, with those present in the UniProt reference cluster 90 (UniRef90) database. The default comparison parameters in HUMAnN 2.0 were set to translated_query_corverage_threshold = 90.0, prescreen threshold_0.01, evalue_threshold = 10, and translated_subject_coverage_threshold = 50.0, and reads of inferior quality were eliminated correspondingly. Then, the protein expression levels in UNniRef90 were quantified as reads per kilobase per million and then compared with the clean reads of each sample to generate a functional database and Kyoto Encyclopedia of Genes and Genomes (KEGG) corresponding to their relative functional abundance. The LEfSe and a Dunn’s test were used to identify the characteristic KEGG metabolic pathways, KEGG. pathways level 1 and KEGG. pathways level 2 in each group. The metabolic pathways were further verified in the MetaCyc database. The Circos diagrams were used to visualize the top 10 characteristic EC enzyme activity pathways in various samples.

### 2.8. Statistical Analysis

The differences in body weight, water intake, and food intake among the groups at each time point were analyzed using a Bonferroni or Tamhane T2 post hoc test or a one-way ANOVA with least-significant difference (Bonferroni) [23], intestinal morphological indicators, histological scores, running distance, concentration of SCFAs in feces, and SCFAs in plasma concentration. In line with the objective of this study, Bray–Curtis dissimilarity was identified as the primary measure to assess the microbial shift caused by antibiotics and various interventions. The most stringent Bonferroni multiple-testing correction was employed for the primary outcome in order to compare the microbial shifts across different groups. The Bray–Curtis was analyzed using permutational multivariate analysis of variance (PERMANOVA). For the diversity and classification relative abundance of the gut microbiome, the metagenomic detection data were analyzed using one-way ANOVA and a Duncan post hoc test according to the literature report (Front Microbiol). For the data that do not meet the normal distribution, a Kruskal–Wallis test was used. *p* < 0.05 indicates statistically significant differences between groups. The statistical analysis was performed using an SPSS 26.0 software. The differences in microbial communities were analyzed using the Bray–Curtis dissimilarity principal component analysis (PCA), and the differences in fecal samples were evaluated using qiime2. Before using the above statistical methods to test the data, normality test is taken to ensure that the statistical method is correct. According to the data obtained from the statistical results, the differences in microbial diversity and different bacterial abundance between the groups can be compared, and the intervention effects of different intervention measures on the gut microbiome of mice can be judged by comparing with the CTL group. According to the PCA analysis, the difference of gut microbiome structure between the other four groups and the CTL group could be determined, and the most ideal intervention effect could be determined.

## 3. Results

### 3.1. Body Weight and Water and Food Intake

Figure 1 and Figure S1 show the effect of different intervention methods on body weight (Figure 1b), food intake (Figure 1c), and water intake (Figure 1d and Figure S1a) in mice. In general, the body weight of each group of mice increased gradually during the experiment, and although it showed some fluctuations, there was no significant difference between the groups at the end of the experiment. There was also no significant difference in total food intake among the groups.

As shown in Figure 1d, compared with the CTL group, the water intake of mice increased significantly when KGM was added, but exercise could offset this increased effect. The EK group significantly reduced the increase in water intake caused by the addition of KGM but still produced a significant difference with the CTL group, the ATBX group, and EXC group. At the end of the experiment, we conducted tests to determine the longest running distance achieved by mice in each group (Figure S1b). This was performed to assess the impact of antibiotics on their sport performance and evaluate the restorative effects of the three interventions. As shown in Figure S1b, even after administering antibiotics, there was a significant improvement in the longest running distance achieved by the exercise intervention group (the EXC and the EK groups) compared with the CTL group; the ATBX group was significantly lower than the CTL group, but there was no significant difference between the KGM group and the CTL group.

### 3.2. Intestinal Morphography

**Figure 2:**
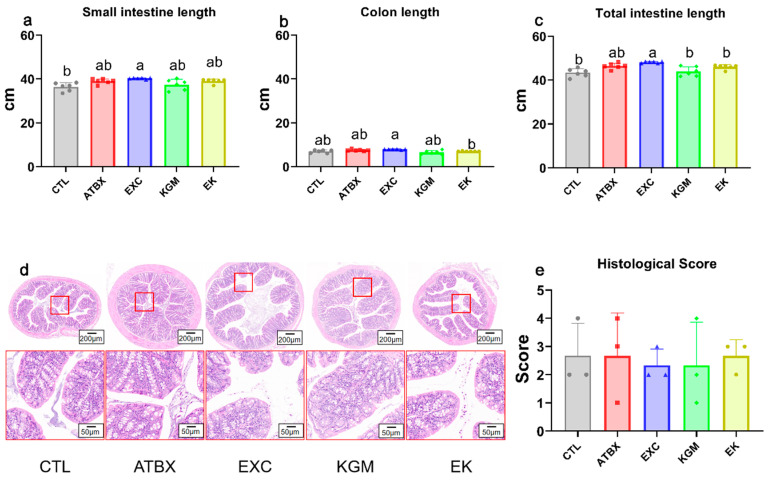
The effects of the different intervention methods on the intestinal tissue of mice. The length of the (**a**) small intestine, (**b**) colon, and (**c**) total intestine, and (**d**) representative pictures of H and E staining and (**e**) histological scores of mice colons. Average of six samples. Error bars represent standard deviation at N = 6 for (**a**–**c**), and N = 3 for (**e**). Different letters indicate the significant difference among different groups for the same index, ANOVA with Bonferroni or Tamhane T2 post hoc test, *p* < 0.05. CTL: control; ATBX: antibiotic; EXC: exercise; KGM: the native KGM; EK: combination of exercise and KGM.

Figure 2a–e show the effects of different interventions on intestinal length and histology. Neither antibiotic intervention nor native KGM supplementation had an effect on colon length. However, compared with the CTL group, the EXC group significantly increased small intestine and whole intestine length. In addition, the H and E staining and histological scores showed no significant changes in colonic morphology among all groups.

### 3.3. Short-Chain Fatty Acids

**Figure 3:**
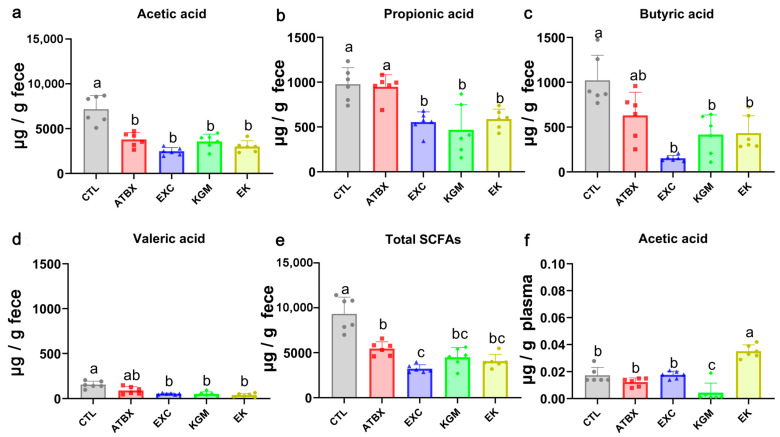
The effect of different intervention methods on SCFAs in mice feces and plasma on day 42. (**a**) Acetic acid, (**b**) propionic acid, (**c**) butyric acid, (**d**) valeric acid, (**e**) total SCFAs, (**f**) acetic acid in plasma. Different letters indicate the significant difference among different groups for the same index, ANOVA with Bonferroni or Tamhane T2 post hoc test, *p* < 0.05. Average of six samples. Error bars represent standard deviation at N = 6. CTL: control; ATBX: antibiotic; EXC: exercise; KGM: the native KGM; EK: combination of exercise and KGM.

Figure 3 shows the generation of SCFAs in feces (Figure 3a–e) and plasma (Figure 3f) in five groups on day 42. The main SCFAs in feces were acetic acid, propionic acid, and butyric acid, and the main SCFA in plasma was acetic acid. Compared with the CTL group, the total contents of SCFAs in the other four groups were significantly reduced, and the content of SCFAs in the EXC group was the lowest, which was significantly different from that in the ATBX group. Specifically, the acetic acid content could not recover to the CTL level after antibiotic perturbation and three intervention methods, and there was significant difference compared with the CTL group. There was no significant difference between the ATBX group and the CTL group in propionic acid, valeric acid, and butyric acid content, and the EXC, KGM, and EK groups did not recover to the CTL group level. In plasma, after the intervention of exercise combined with KGM (the EK group), the content of acetic acid was increased, and there was a significant difference compared with the other four groups. After supplementing KGM (the KGM group), the content of acetic acid in the plasma of the mice was lower than that in the CTL, ATBX, EXC, and EK groups and showed a significant difference.

### 3.4. Gut Microbiome (16S rRNA Sequencing\Bioinformatic Analysis)

In general, both exercise and KGM intervention led to significant changes in the gut microbiome. Table 2 shows the gut microbiome α diversity index on day 42. The diversity index is an analysis of species diversity in a sample, including the richness and evenness of species composition in the sample. Indices such as Observed OTUs and Shannon and Faith Phylogenetic Diversity (Faith_pd) are usually used to assess the species diversity of a sample, and the higher the index is, the more complex the diversity of the sample is. A chao1 index is an index used to reflect species richness (number of species). It extrapolates from the observed results a theoretical richness that is closer to the true richness. The observed OTUs index refers to the number of OTUs actually measured in the sample and the index that measures the richness of OTUs in the sample. The Shannon index, which is calculated taking into account the total number of OTUs in the sample and the proportion of each OTU. The Faith_pd is a diversity index calculated based on a phylogenetic tree, which uses the representative sequences of OTUs in individual samples to calculate the distance to build a phylogenetic tree, and adds the values of all the representative sequences in a sample to get the values. The Simpson index is used to estimate the similarity of the community, and it reflects the diversity based on accounting proportion of species in the community [24].

The chao1 and observed_otus results were similar; only the EK group recovered to the CTL group level, while the ATBX group, EXC group, and KGM group were significantly lower than the CTL group, and the KGM group was significantly lower than the ATBX group and EXC group. The Faith_pd index of the ATBX group, EXC group, KGM group, and EK group decreased significantly compared with the CTL group, and the KGM group was significantly lower than the EK group. There was no significant difference between Shannon and Simpson among all groups.

### 3.5. Gut Microbiota

**Figure 4:**
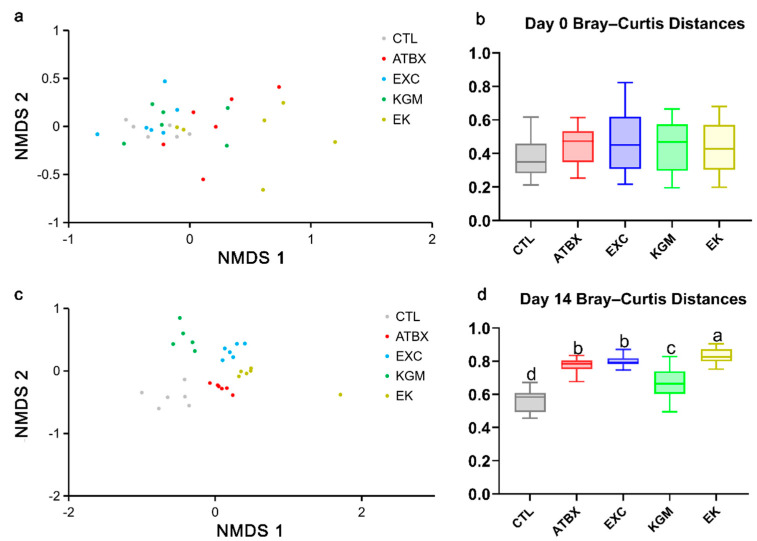
Effects of different intervention methods on fecal microbial composition of mice on day 0 and day 14: (**a**) the principal component analysis (PCoA) on day 0; (**b**) the Bray−Curtis distances from five groups to CTL group on day 0; (**c**) the principal component analysis (PCoA) on day 14; (**d**) the Bray−Curtis distances from five groups to CTL group on day 14. Not having the same letters indicates the significant difference among different groups for the same index. CTL: control; ATBX: antibiotic; EXC: exercise; KGM: the native KGM; EK: combination of exercise and KGM.

On day 0, as depicted in Figure 4a,b and Table S4, only minimal differences in gut microbial composition were observed within each group. The Table S3 presents the relative abundance of the top 20 taxa at different levels on day 0 and also showed no significant differences in the Bray–Curtis distance among all groups, indicating good comparability at the beginning of the experiment. 

On day 14, as illustrated in Figure 4c,d and Table S5, there was a significant increase in Bacteroidetes at the phylum level for both the EXC group (77.64%) and the EK group (73.57%) compared with the CTL group (52.70%). In contrast, the relative abundance of Verrucomicrobia in the EXC group (0.31%) was significantly lower compared with the CTL group (13.53%). Additionally, compared with the CTL group (3.40%), the relative abundance of Proteobacteria in the KGM group (0.33%) and the EK group (0.53%) was significantly reduced. At the family level, compared with the CTL group (13.53%), the relative abundance of Verrucomicroaceae in the EXC group (0.31%) and the EK group (2.01%) decreased significantly. The relative abundance of Paraprevotellaceae in the EK group (17.77%) was significantly higher than that in the CTL group (4.6%). However, the relative abundances of Bifidobacteriaceae (4.10% with the CTL versus 0.04% with KGM versus 0.02% with EK) decreased, while Alcaligenaceae (0.70% with the CTL versus 0.12% with KGM versus 0.45% with EK) and Prevotellaceae (0.69% with CTL versus 0.00% with KGM versus 0.71% with EK) showed a reduction due to KGM supplementation. At the genus level, exercise single intervention increased the relative abundances of unclassified taxa (43.83% with CTL versus 72.45% with EXC) but decreased *Akkermansia* (13.53% with CTL versus 16.67% with EXC) and *Parabacteroides* (4.14% with CTL versus 1.19% with EXC). Compared with the CTL group, the relative abundance of *Helicobacter* (2.48% with CTL versus 0.00% with KGM), *Sutterella* (0.69% with CTL versus 0.12% with KGM), and *Prevotellae_prevotella* (0.69% with CTL versus 0.00% with KGM) in the KGM group was significantly decreased. The relative abundance of *Akkermansia* (13.53% with CTL versus 2.06% with EK), *Helicobacter* (2.48% with CTL versus 0.17% with EK), and *Erysipelotrichacea_Clostridium* (0.74% with CTL versus 0.05% with EK) in the EK group also decreased significantly. In terms of the Bray–Curtis distance, the KGM, EXC, EK, and ATBX groups had significant differences compared with the CTL group.

**Figure 5:**
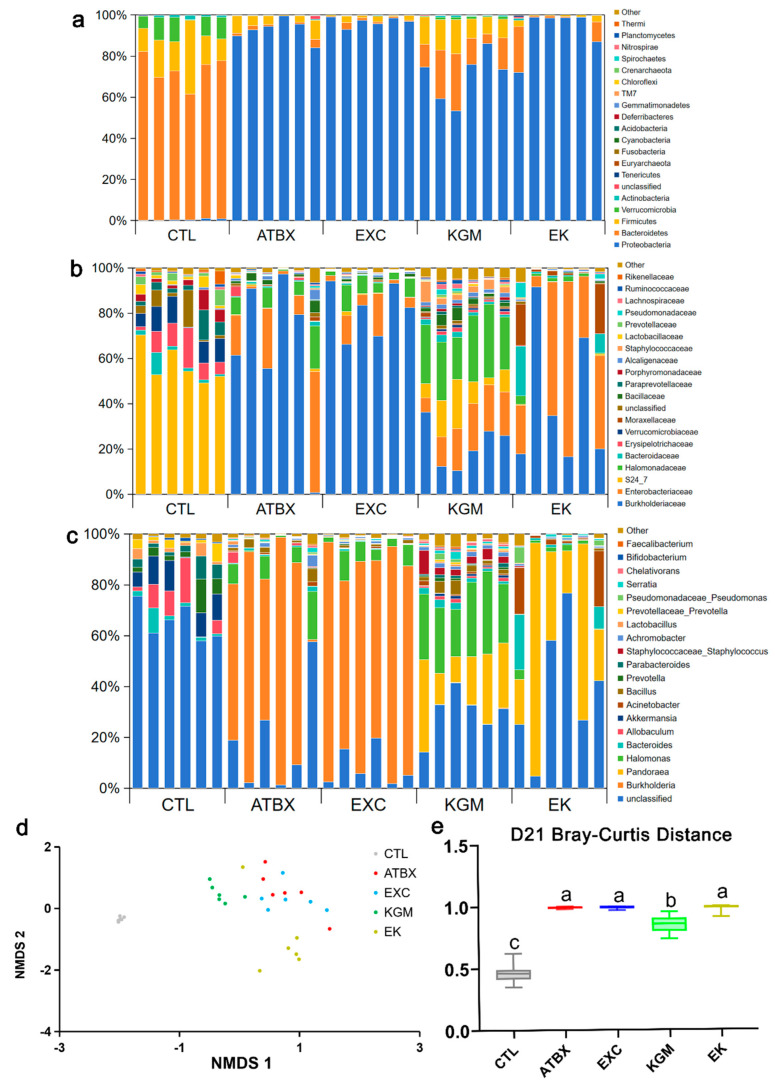
Effects of different intervention methods on fecal microbial composition of mice on day 21: (**a**) at phylum level, (**b**) at family level, and (**c**) at genus level. (**d**) The principal component analysis (PCoA), (**e**) Bray−Curtis distances from five groups to CTL group. Samples (N = 30) were colored by treatment. Not having the same letters indicates the significant difference among different groups for the same index. CTL: control; ATBX: antibiotic; EXC: exercise; KGM: the native KGM; EK: combination of exercise and KGM.

On day 21, as illustrated in Figure 5a–e and Table S6, one-week perturbance of antibiotic resulted in more pronounced alterations in the gut microbiota of other four groups compared with the CTL group. At the phylum level, antibiotic intervention resulted in a decrease in the relative abundance of Bacteroidetes (72.83% with CTL versus 1.54% with ATBX), Firmicutes (17.31% with CTL versus 5.08% with ATBX), Verrucomicrobia (8.17% with CTL versus 0.05% with ATBX), Actinobacteria (0.99% with CTL versus 0.18% with ATBX), Tenericutes (0.13% with CTL versus 0.01% with ATBX), and *TM7* (0.01% with CTL versus 0.00% with ATBX) but an increase in Euryarchaeota (0.00% with CTL versus 0.07% with ATBX) compared with the CTL group. The KGM group partially mitigated these changes by preserving higher levels of Bacteroidetes (72.83% with CTL versus 1.54% with ATBX versus 15.85% with KGM), Firmicutes (17.31% with CTL versus 5.08% with ATBX versus 11.98% with KGM), and Verrucomicrobia (8.17% with CTL versus 0.05% with ATBX versus 0.87% with KGM), However, exercise intervention (EXC group and EK group) had no significant preventive effect on antibiotic-induced microbiome changes; the relative abundance of microbiome in the EXC group and the EK group had no significant difference compared with the ATBX group at the phylum level. At the family level (Figure 5b), compared with the CTL group, antibiotic intervention resulted in a decrease in the relative abundances of S24_7 (57.04% with CTL versus 0.41% with ATBX), Erysipelotrichaceae (8.64% with CTL versus 0.98% with ATBX), Verrucomicrobiaceae (8.17% with CTL versus 0.05% with ATBX), unclassified (5.12% with CTL versus 0.58% with ATBX), Paraprevotellaceae (4.78% with CTL versus 0.12% with ATBX), Porphyromonadaceae (3.78% with CTL versus 0.05% with ATBX), Lactobacillaceae (2.45% with CTL versus 0.35% with ATBX), Prevotellaceae (2.83% with CTL versus 0.16% with ATBX), and Rikenellaceae (1.56% with CTL versus 0.03% with ATBX). In contrast, there was an increase in the relative abundances of Pseudomonadaceae (0.00% with CTL versus 0.28% with ATBX). However, there was no significant difference between the KGM group and the CTL group. The Pseudomonadaceae in the EXC group (0.78%) also had no significant difference compared with the CTL group. At the genus level (Figure 5c), antibiotic intervention caused reductions in *Allobaculum* (7.27% with CTL versus 0.97% with ATBX), *Akkermansia* (8.17% with CTL versus 0.05% with ATBX), *Prevotella* (4.78% with CTL versus 0.09% with ATBX), *Parabacteroides* (3.78% with CTL versus 0.04% with ATBX), *Lactobacillus* (2.45% with CTL versus 0.35% with ATBX), *Prevotella_aceaeprevotella* (2.83% with CTL versus 0.16% with ATBX), *Serratia* (0.00% with CTL versus 0.54% with ATBX), and *Bifidobacterium* (0.64% with CTL versus 0.06% with ATBX). On the other hand, antibiotics led to an increase in *Pseudomonas* (0.00% with CTL versus 0.28% with ATBX) and *Faecalibacterium* (0.00% with CTL versus 0.25% with ATBX). Notably, KGM intervention effectively attenuated most of these changes. As shown in Figure 5d, among the four groups with antibiotic perturbance, compared with the CTL group, the Bray–Curtis distances of the ATBX group, the EXC group, the KGM group, and the EK group were significantly different, but the Bray–Curtis distance of KGM group was closer to that of the CTL group and had a significant difference compared with the ATBX group, the EXC group, and the EK group.

**Figure 6:**
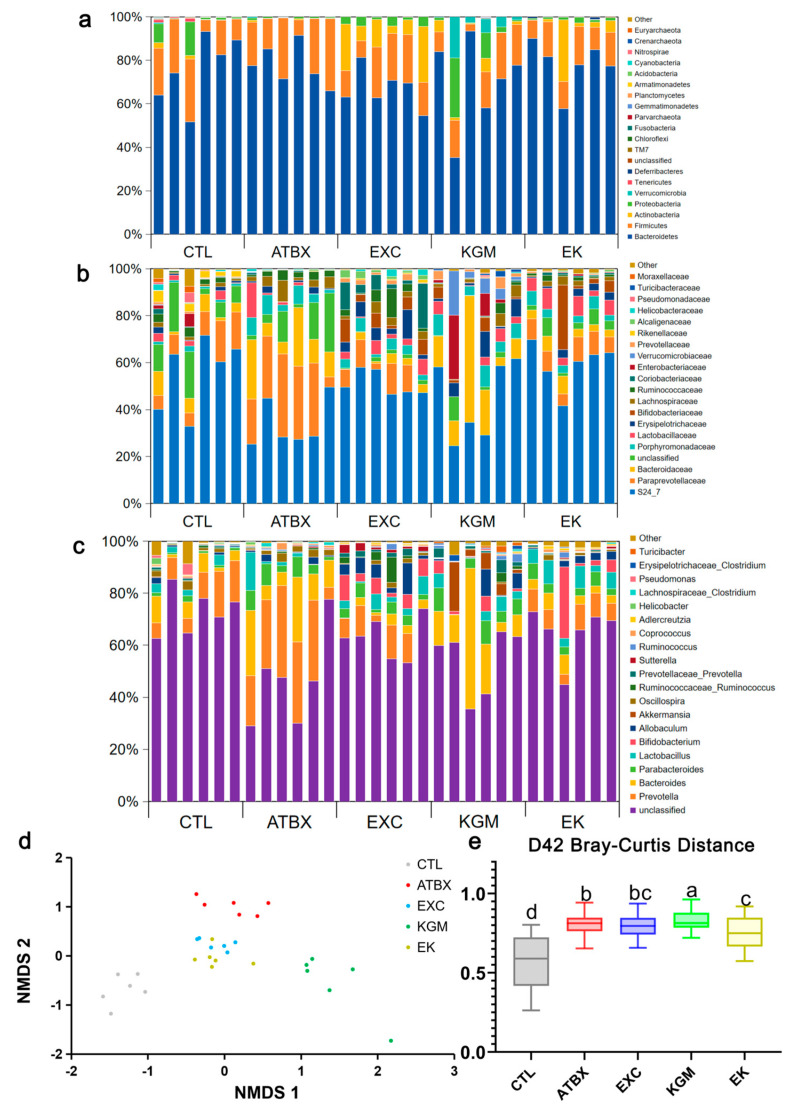
Effects of different intervention methods on fecal microbial composition of mice on day 42: (**a**) at phylum level, (**b**) at family level, and (**c**) at genus level. (**d**) The principal component analysis (PCoA), (**e**) Bray−Curtis distances from five groups to the CTL group. Not having the same letters indicates the significant difference among different groups for the same index. CTL: control; ATBX: antibiotic; EXC: exercise; KGM: the native KGM; EK: combination of exercise and KGM. Samples (N = 30) were colored by treatment.

As illustrated in Figure 6a–e and Table S7, significant differences were observed in the relative abundance of Tenericutes (0.45% with CTL versus 0.00% with ATBX versus 0.05% with EK) at the phylum level on day 42 when comparing the ATBX group with the CTL group. However, the combination of exercise and the KGM restored these differences to a non-significant level, similar to that of the CTL group. There was no significant difference between the EXC group and the KGM group compared with ATBX group. At the family level (Figure 6b), compared with the CTL group, there is a significant difference in the relative abundance of Bacteroidaceae (3.73% with CTL versus 14.23% with ATBX), Rikenellaceae (1.96% with CTL versus 0.09% with ATBX), and Helicobacteraceae (0.11% with CTL versus 0.61% with ATBX) in the ATBX group, while there is no significant difference in the relative abundance of Bacteroidaceae (3.73% with CTL versus 14.23% with ATBX versus 7.30% with EXC versus 4.46% with EK) in the EK group and EXC group compared with the CTL group. The relative abundance of Rikenellaceae (1.96% with CTL versus 0.09% with ATBX versus 0.49% with EXC) in the EXC group was not significantly different from that in the CTL group. The addition of KGM (KGM group, EK group) showed no significant difference in relative abundance of Helicobacteraceae (0.11% with CTL versus 0.12% with KGM versus 0.00% with EK) compared with the CTL group. At the genus level (Figure 6c), compared with the CTL group, the relative abundance of unclassified (79.72% with CTL versus 45.27% with ATBX), *Bacteroides* (3.73% with CTL versus 14.23% with ATBX), *Prevotellaceae_Prevotella* (0.33% with CTL versus 0.00% with ATBX), *Lachnospiraceae_Clostridium* (0.01% with CTL versus 0.45% with ATBX), and *Pseudomonas* (0.77% with CTL versus 0.00% with ATBX) in the ATBX group had significant differences. However, the relative abundance of the unclassified (79.72% with CTL versus 65.07% with EK), *Bacteroides* (3.73% with CTL versus 4.46% with EK), *Prevotellaceae_Prevotella* (0.33% with CTL versus 0.30% with EK), and *Lachnospiraceae_Clostridium* (0.01% with CTL versus 0.14% with EK) in the EK group had no significant difference compared with the CTL group. FIG S1C represents the ratio of Firmicutes/Bacteroidetes (F/B) at each time point. The results obtained were similar to those described previously, with antibiotic intervention leading to large fluctuations in the gut microbiota on day 21, and KGM had the best protective effect compared with other interventions. On day 42, there was no significant difference in the F/B ratio among the groups. As shown in Figure 6d, compared with the CTL group, the Bray–Curtis distance of the other four groups was significantly different, and the Bray–Curtis distance between the KGM group and the CTL group was the farthest, which indicates that the species composition and structure were the most different. There was a significant difference between the EK group and the ATBX group (*p* = 0.04, η^2^ = 0.43), indicating that the recovery effect of the EK group was better than the ATBX group.

**Figure 7:**
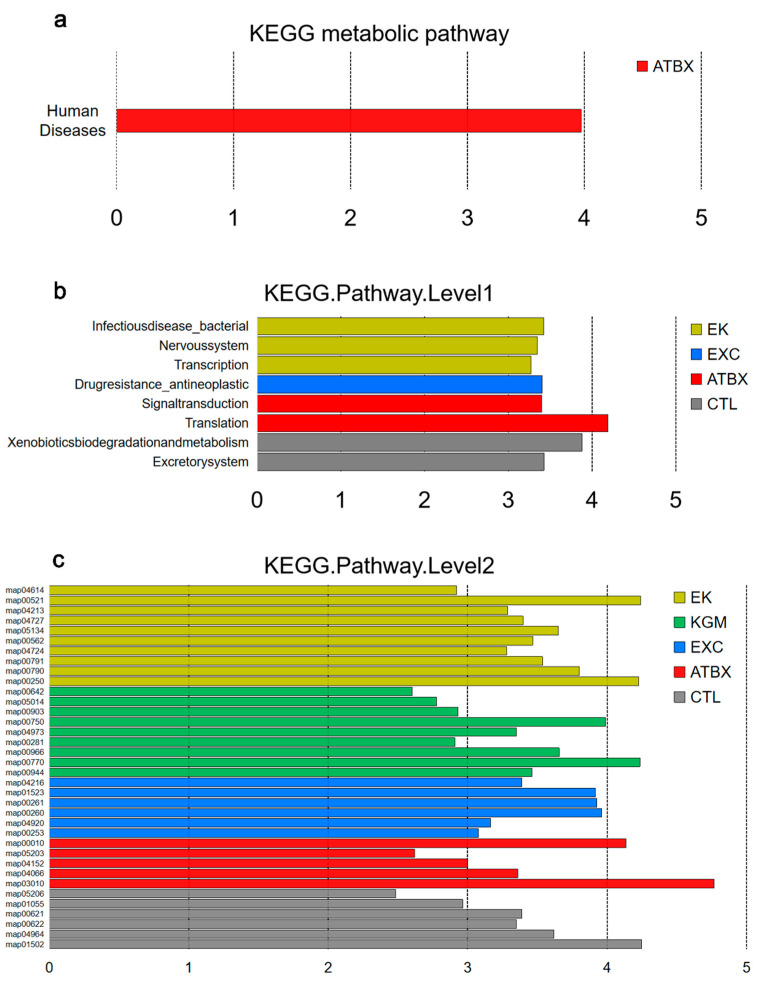
Effects of different intervention methods on KEGG metabolic pathway in mice at day 42: (**a**) KEGG metabolic pathways characteristics of CTL, ATBX, EXC, KGM, and EK groups at LDA2 level were analyzed via LEfSe, (**b**) KEGG. pathways level 1 characteristics of CTL, ATBX, EXC, KGM, and EK groups at LDA2 level were analyzed by LEfSe (**c**) KEGG. pathways level 2 characteristics of CTL, ATBX, EXC, KGM, and EK groups at LDA2 level were analyzed by LEfSe. Samples (N = 15) were colored by treatment. CTL: control; ATBX: antibiotic; EXC: exercise; KGM: the native KGM; EK: combination of exercise and KGM.

As depicted in Figure 7a–c, on the KEGG level 1, “Human Diseases” were enriched in the ATBX group. On the KEGG level 2 (Figure 7b), antibiotic supplementation increased “Translation” and “Signal transduction”, while exercise increased “Drug resistance_antineoplastic”. The combined intervention of exercise and KGM (EK group) improved the LDA score of “Infectious disease_bacterial”, “Nervous system”, and “Transcription”.

At the pathway level (Figure 7c), the intervention of ATBX significantly enriched multiple KEGG pathways, including Ribosome (map03010), Glycolysis/Gluconeogenesis (map00010: KO0850, K00001), HIF-1 signaling pathway (map04066), viral carcinogenesis (Map05203), and AMPK signaling pathway (map04152: K000850).

The EXC group mainly enriched biosynthesis and energy metabolism pathways, such as Monobactam biosynthesis (map00261: K01714); Antifolate resistance (map01523: K00560); Glycine, serine, and threonine metabolism (map00260: K00382); Tetracycline biosynthesis (map00253: K18221); and Adipocytokine signaling pathway (map04920: K01897).

In the KGM group, there was enrichment in biosynthesis- and glucose-metabolism-related pathways like Pantothenate and CoA biosynthesis (map00770), Glucosinolate biosynthesis (map00966), Flavone and flavonol biosynthesis (map00944), and Vitamin B6 metabolism (map00750), along with Carbohydrate digestion and absorption (map04973). The combined intervention of exercise and KGM resulted in enrichment of Streptomycin biosynthesis (map00521: K01858), Alanine, aspartate and glutamate metabolism (map00250: K00265, K01915), Folate biosynthesis (map00790), Inositol phosphate metabolism (map00562), and Glutamatergic synapse (map04724: K01915) among other metabolic pathways.

**Figure 8:**
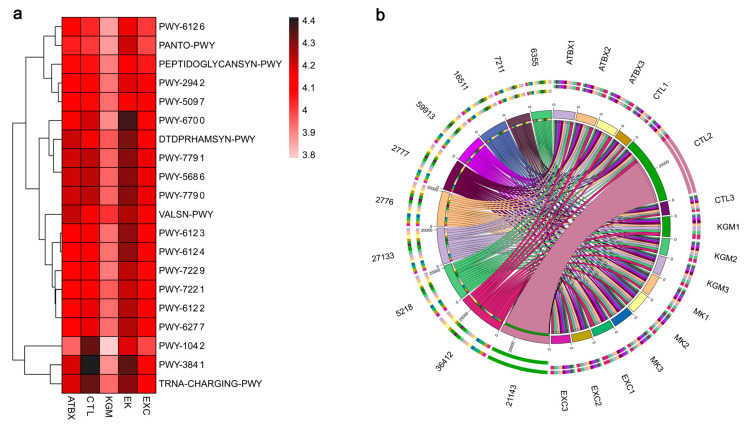
Effects of different intervention methods on MetaCyc metabolic pathway in mice at day 42. (**a**) MetaCyc metabolic pathway heat map after different intervention methods. (**b**) As shown in the Circos diagram, genes for enzyme activity were altered in five groups (Enzyme nomenclature database). CTL: control; ATBX: antibiotic; EXC: exercise; KGM: the native KGM; EK: combination of exercise and KGM. Samples (N = 15) were colored by treatment.

In addition, the differential metabolic pathways in the MetaCyc database are displayed as heat maps (Figure 8a). For the ATBX group and the EK group, UMP biosynthesis III (PWY-7791), UMP biosynthesis I (PWY-5686), UMP biosynthesis II (PWY-7790), folate transformations II (PWY-3841), and TRNA-CHARGING-PWY were not significantly different compared with the CTL group. The inosine-5’-phosphate biosynthesis I (PWY-6123), inosine-5’-phosphate biosynthesis II (PWY-6124), 5-aminoimidazole ribonucleotide biosynthesis II (PWY-6122), superpathway of 5-aminoimidazole ribonucleotide biosynthesis (PWY-6277), guanosine ribonucleotides de novo biosynthesis (PWY-7221), and other pathways related to purine metabolism were enriched in the EK group.

The Circos diagram (Figure 8b) shows the proportion of each EC enzyme activity (top 10 abundance) in each sample, and the proportion of each EC enzyme activity in each sample. The ATBX group and the EK group DNA-directed DNA polymerase (EC 2.7.7.7.), DNA topoisomerase-ATP-hydrolyzing (EC 5.99.1.3.), and 6-phosphofructokinase (EC 2.7.1.11), restoring them to the CTL level, but the EXC group and the KGM group had significant differences compared with the CTL groups. In addition, except for the KGM group, beta-galactosidase (EC 3.2.1.23) in ATBX group, EXC group, and EK group had no significant difference compared with the CTL group.

### 3.6. Correlation Analysis

**Figure 9:**
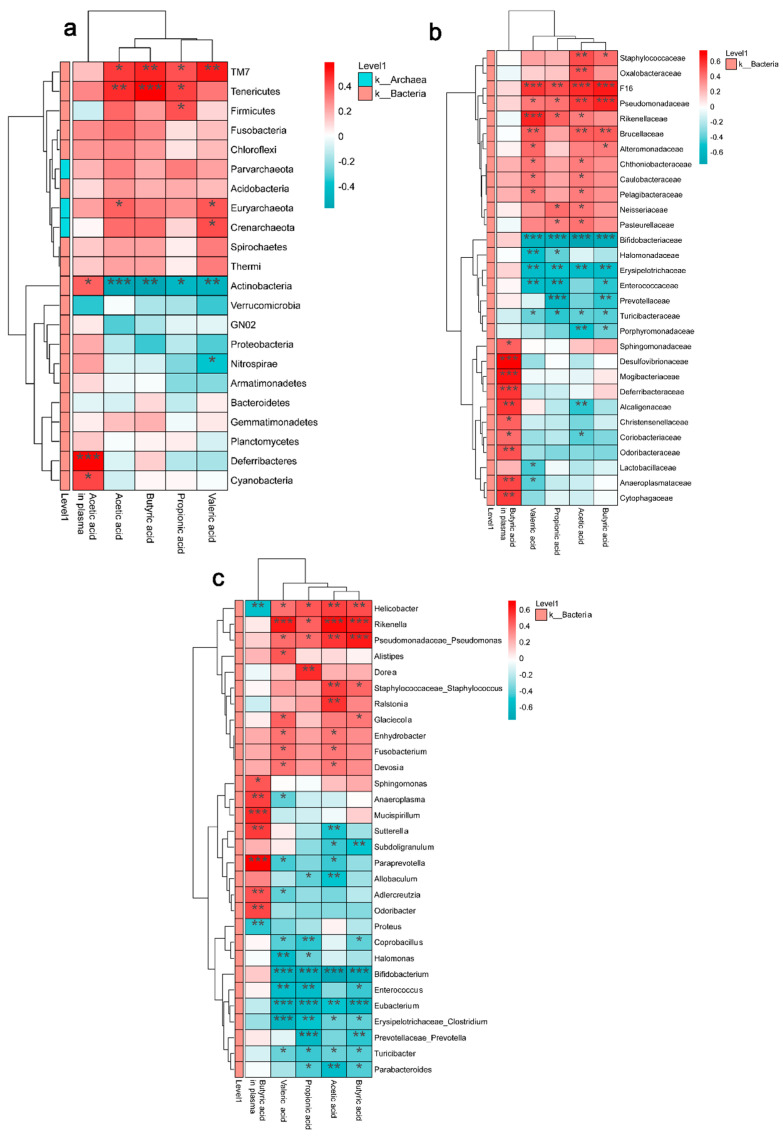
A correlation heatmap linking the top 20 significantly differentiated bacteria with the SCFAs: (**a**) phylum level; (**b**) family level; and (**c**) genus level. Samples (N = 15) were colored by treatment. * 0.01 ≤ *p* < 0.05, ** 0.001 ≤ *p* < 0.01, *** *p* < 0.001. CTL: control; ATBX: antibiotic; EXC: exercise; KGM: the native KGM; EK: combination of exercise and KGM.

At the end of the experiment, the correlation between the relative abundance of different levels of gut microbiome and SCFAs was analyzed. The heat maps (Figure 9a) show that at the phylum level, Actinobacteria is significantly negatively correlated with acetic acid, propionic acid, butyric acid, and Valeric acid, while Tenericutes and TM7 are significantly positively correlated with acetic acid, propionic acid, and butyric acid. In addition, Actinobacteria, Deferribacteres, and Cyanobacteria are positively correlated with acetic acid levels in plasma. At the genus level (Figure 9c), *Prevotella, Parabacteroides, Bifidobacterium, Coprococcus,* and *Erysipelotrichaceae_Clostridium* are significantly negatively correlated with butyric acid. *Helicobacter* is significantly positively correlated with butyric acid. *Paraprevotella*, *Sutterella*, and *Adlercreutzia* are significantly positively correlated with acetic acid level in plasma, and *Helicobacter* are significantly negatively correlated with acetic acid level in plasma. Meanwhile, an increase in *Adlercreutzia* and a decrease in *Helicobacter* were observed in the EK group, with a similar trend seen in the EXC group.

## 4. Discussion

In our previous research, we found that compared with the lower molecular weight KGMs, the native KGM with higher molecular weight can more effectively prevent and counteract the perturbation of the gut microbiome caused by antibiotics [9,25]. Additionally, compared with both higher and lower concentrations, the moderate concentration of KGM (2.5 g/L in drinking water) was found to be more effective in alleviating the side effects of excessive exercise on the gut microbial composition and function [20]. Therefore, in this experiment, we employed a strategy of free access to water supplemented with 2.5 g/L of native KGM for mice. In this experiment, we found that the combination intervention using this representative concentration exhibited the best effect in counteracting dysbiosis. It is important to consider more concentrations in future studies.

In the present study, the average daily water intake in the KGM group (10.33 g/day/mice) was significantly higher than that in the CTL group (4.76 g/day/mice) (Figure 1d). Specifically, each mouse in the KGM group consumed approximately 1000 mg/kg BW of KGM per day, while mice in the EK (7.49 g/day/mice) group consumed around 750 mg/kg BW of KGM per day. The increase in water intake in the KGM group was consistent with our previous study [20], and we observed that exercise partially counteracted this trend, although there was still a significant difference compared with water intake in the CTL group. And a study has also reported that the amount of water that mice drink may fluctuate due to many factors [26]. This led to differences in dietary fiber intake between the KGM group and the EK group. Although this issue may affect the comparability between the two groups, we can still comprehensively consider the final outcome while taking into account the influence of KGM on water intake as a confounder factor. To improve this, to address this issue and improve the study, future research should consider isolating the effect of adding exercise while maintaining the same dietary fiber concentration.

Some studies have reported varied results regarding the recovery time following antibiotic perturbation, as a result of the influence of various factors (animal models, environment, antibiotic cocktail formulations, etc.) on this process [5,27,28]. A study indicated that it takes about four weeks for the gut microbiota to recover to some extent after antibiotic treatment [29]. However, another study showed that the recovery time of four weeks post-antibiotic perturbance was still far from reaching the fecal alpha diversity and bacterial richness of the control group [30]. In our previous study, we found that a two-week period of natural recovery following antibiotic perturbance resulted in a limited level of improvement, while the supplementation of singular KGM significantly accelerated this process [9]. Therefore, considering both of the test cost and effects, the present study employed a compromised three-week recovery period to investigate the extent of natural recovery in mice during this time and to determine if any interventions can accelerate the recovery process. The results showed (Table 2) that although there was a certain degree of recovery in alpha diversity, bacterial richness, and beta diversity in mice, the effect was more pronounced after exercise or exercise combined with KGM intervention. As far as we know, this is the first time that dysbiosis induced by antibiotics has been addressed with exercise in vivo.

Following a two-week intervention with exercise (prior to the administration of antibiotics), an increase in the relative abundance of Bacteroides in the feces of mice was observed in the EXC group and the EK group (Table S4). Bacteroides is the main phylum for digestion of dietary fiber [31], and it is able to convert carbohydrates into usable nutrients; it is widely recognized to be positively correlated with a healthier metabolic status [32]. Bacteroides were also inversely associated with type 2 diabetes in humans [33]. This is consistent with previous studies [33]; the changes in EXC and EK groups are mainly caused by exercise, and the main reason for the changes may be due to the positive effect of exercise on promoting intestinal health; the changes in individual metabolic characteristics caused by exercise also lead to this result. Additionally, Bacteroidetes have been reported to play a role in the processing of complex carbohydrates [30,32]. This may also explain the increase induced by exercise, as more energy is required under such conditions. Meanwhile, the two weeks of KGM intervention resulted in a decrease in the Proteobacteria phylum, some Proteobacteria species may be associated with intestinal barrier dysfunction, chronic inflammation, and insulin resistance and also may be associated with the severity of some diseases [34].

However, after one week of antibiotic intervention (at the end of third week), the addition of KGM effectively mitigated the antibiotic-induced changes in microbiota better than the EXC group (Figure 5e). This finding further supports the protective effects of KGM, which is consistent with our previous finding [9]. After analysis and comparison, the F/B ratio of the four time points can also support this conclusion. In order to comprehensively observe the changes caused by antibiotics and to exclude other confounding factors that may affect the experimental results, histological scoring was conducted on colon tissues. The experimental results were consistent with our previous studies, and no significant differences were found between groups [9]. It is noteworthy that exercise alone or combined with KGM intervention did not exhibit significant preventive effects. Exercise leads to blood redistribution to the muscles, causing ischemia in the gastrointestinal tract, which can result in gastrointestinal disorders [35], and this may lead to a prolonged stay or an increased absorption of antibiotics in the gastrointestinal tract and an increased risk of harm to the body. Meanwhile, antibiotic intervention also reduces exercise capacity [18], and inappropriate exercise load can lead to decreased immunity, ultimately resulting in the occurrence of an “Open Window” phenomenon [36]. Considering this complex dynamic interaction, we have chosen a low–moderate intensity of exercise. Several studies have also reported a relationship between exercise and antibiotics. Doxorubicin is an effective chemotherapy antibiotic utilized in cancer treatment. Previous studies have demonstrated that a 4- to 12-week preconditioning regimen of treadmill exercise can mitigate its side effects (liver toxicity or cardiac injury) [37,38], while another study reported that 10-day exercise preconditioning has a limited effect on doxorubicin-induced tissue toxicity [39]. In our previous study, supplementing KGM for two weeks and intervening with antibiotics at the same time can achieve a good preventive effect. Therefore, in this experiment, the time of exercise preconditioning was also decided to be two weeks, and exercise training was still carried out within the weeks of antibiotic intervention, which is different from the exercise preconditioning method mentioned above. This may also explain why exercise is less effective in prevention. Therefore, the timing of exercise as a preventive measure holds significant importance; inappropriate timing may exacerbate the adverse effects caused by antibiotics. The limited preventive effect observed in the EXC and the EK groups may be a result of a combination of factors (low–moderate exercise intensity, increased of absorption antibiotics, and timing of exercise). According to some of the literature mentioned above, in the later experiments, stopping the exercise intervention in mice during antibiotic intervention, which might get different results. In addition, in future studies, the exercise intensity of the intervention mice will be gradually increased to determine the different effects of different exercise regimens.

At the end of the experiment (on day 42), although the composition of gut microbiome in the stool of the ATBX group was partially recovered, the gut microbial composition in the EK group was closer to that of the CTL group from the perspective of species structure (Figure 6e). Specifically, antibiotic intervention significantly decreased the relative abundance of Tenericutes phylum and unclassified taxa; however, only the EK group could restore these levels to the CTL level (Table S6). Previous studies have reported lower levels of Tenericutes in elderly patients with type 2 diabetes and obese individuals with metabolic dysfunction [40,41]. Furthermore, while there was no significant difference in the relative abundance of Verrucomicrobia between the KGM group and the CTL group after one-week of antibiotic intervention, showed a significantly higher abundance of Verrucomicrobia was observed in the KGM group compared with the CTL group after three-week recovery. This increase was also observed within specific taxa, such as Verrucomicrobiaceae and *Akkermansia,* which belong to the Verrucomicrobia phylum. *Akkermansia* has been identified as a promising probiotic candidate for improving indicators related to type 2 diabetes both in mice and humans [42], and it has been demonstrated to upregulate genes involved in maintaining intestinal barrier function, thereby promoting intestinal homeostasis [43]. Additionally, a higher relative abundance of *Akkermansia* is often associated with a healthier metabolic status among adults, as it correlates negatively with body fat mass and glucose intolerance [44]. Consistent with previous findings from other studies [45], our results indicate that supplementation with KGM can effectively increase the relative abundance of *Akkermansia*.

In the current study, the KGM group demonstrated a superior preventive effect on day 21 compared with the two groups with exercise intervention. In contrast, the EK group exhibited the most effective restorative effect on day 42 among the three intervention groups. Our previous study reported that KGM with high viscosity could adsorb antibiotics, which partially contribute to the protective effect on gut bacteria [46]. However, whether the presence of KGM affects the therapeutic efficacy of antibiotics is still doubted. For patients, antibiotics are essential for treating inflammation and infections, but it is also important to consider the balance of gut microbiota. In other words, we aim to minimize harm to the gut microbiome while maximizing the effectiveness of antibiotics. If the use of KGM for prevention compromises the efficacy of antibiotics, then it is not primarily recommended. However, further experiments are required to confirm this. From this perspective, exercise may be a more effective preventive strategy and also help to avoid any potential impact on the efficacy of antibiotics. However, the increased energy requirement during exercise enhances the metabolism and utilization of KGM by gut microbiota [47], consequently reducing the concentration in the gut and decreasing the absorption of antibiotics. These factors may explain why the preventive effect in the EK group was inferior to that in the KGM group at day 21. On day 42, despite natural recovery over 3 weeks, the gut microbiome was still not fully restored (CTL vs. ATBX). However, exercise continuously regulated the gut microbiome through energy homeostasis and restored microbial composition, especially with sufficient usable carbohydrate substrates (for example, KGM). As a result, the EK group better reduced the impact induced by antibiotics compared with singular KGM intervention. Therefore, the recovery effect of the EK group was better than that of both the EXC and KGM groups on day 42.

We found that the concentration of acetic acid in plasma was significantly increased in the EK group (Figure 3f). Studies reported that the antibiotic treatment reduced the cross-sectional area of muscle fibers, and acetic acid supplementation could counteract this negative effect [48]. Acetic acid in plasma concentration was significantly increased in the EK group compared with other four groups, suggesting that the combination of exercise and KGM intervention may be able to better utilize acetic as an energy source [49], even at the same fecal acetic concentration. In addition, two weeks of exercise led to an increase in the relative abundance of many SCFA-producing bacteria (such as Bacteroides, Prevotella, and so on), but this effect was offset after perturbance of antibiotics, and failed to fully recover even after a three-week recovery period. The correlation analysis between SCFAs and the relative abundance of gut microbiome (Figure 9c) showed that *Adlercreutzia* levels were significantly positively correlated with the acetic acid level in plasma, and *Helicobacter* levels were significantly negatively correlated with the acetic acid level in plasma. Meanwhile, at the experiment end, the EK group was *Adlercreutzia*-increased and *Helicobacter*-decreased. A recent study identified *Adlercreutzia* as a protective bacterium for Alzheimer’s disease [50], while *Helicobacte* was highly associated with chronic gastritis incidence [51]. This suggests that exercise combined with KGM intervention can not only increase the acetic content in plasma but also may have a positive effect on the body by altering the gut microbiome.

Additionally, the EK group was enriched with a variety of purine-metabolism-related pathways (Figure 8a). Some gut microbiomes can use purines as carbon and energy sources anaerobically, consequently affecting the host’s purine homeostasis [52]. A study demonstrated that antibiotic intervention disrupted the gut microbiota of mice, resulting in elevated serum uric acid levels and disturbed purine metabolism [53]. Some animal studies reported that *Lactobacillus* can reduce the serum uric acid level of high-uric-acid animal models [54,55]. In the present study, there was no significant difference in *Lactobacillus* levels between the KGM group and the CTL group on day 21; the relative abundance of *Lactobacillus* in the ATBX, EXC, and EK groups was significantly decreased compared with the CTL group. At the experiment end, the relative abundance of *Lactobacillus* in the EK group was significantly higher than that in the CTL group (Table S6). Based on these results and the Bray–Curtis distance (Figure 6e), exercise combined with KGM intervention can not only promote the recovery of gut microbiome but may also regulate uric acid levels by increasing the relative abundance of *Lactobacillus*. Moreover, these two effects may also contain an interaction [56]. Previous studies have demonstrated that nucleotide metabolism actively promotes antibiotic-induced bacterial death [57], which may also provide a possible explanation for the limited efficacy of the exercise–KGM combination in preventing antibiotic disruption. Furthermore, a study has reported a significant increase in the presence of bacteria containing purine-metabolizing proteins (*Bifidobacterium bifidum* and *Ruminococus gnavus*) in the feces of mice with high uric acid. Conversely, there was a notable decrease in the presence of bacteria-containing proteins associated with purine-recycling pathways (*Lactobacillus vaginalis* and *Allobaculum*) [53]. In the current study, there was a trend of higher relative abundance of *Allobaculum* in the EXC, KGM, and EK groups compared to the CTL group. However, this difference was not statistically significant, possibly due to the short duration of exercise. This suggests that single exercise and KGM supplement may also have a regulatory effect on the serum uric acid level induced by antibiotics. However, the measurement of uric acid excretion was not applicable in the present study, due to the lack of urine collection. Further studies are necessary to confirm the relevant characteristics and clarify the relationships.

## 5. Conclusions

In conclusion, the combination of exercise and KGM demonstrated a more effective reversal of the damage caused by antibiotics on gut microbiome compared with two singular interventions. It is worth noting that supplementing KGM showed the best preventive effect, while exercise exacerbated the dysbiosis immediately after antibiotic perturbation. Additionally, the combination of exercise and KGM exhibited a certain degree of regulatory effect on purine metabolism, but the causal relationship between restoring the species structure of the gut microbiome community and regulating purine metabolism still needs further research. In summary, the present study provides a promising solution and research direction for preventing and regulating dysbiosis caused by antibiotics or even other factors. Further studies are required to investigate the timing and broader impact of exercise on gut microbial metabolites.

## Figures and Tables

**Table 1:** The protocol of the exercise program.

Week	Velocity (m/min)	Duration (min)
1	13	30
2, 3	13	40
4, 5, 6	14	40

**Table 2:** The alpha-diversity indices of feces of mice on day 42. CTL: control; ATBX: antibiotic; EXC: exercise; KGM: the native KGM; EK: combination of exercise and KGM.

Groups.	Chao1	Faith_pd	Observed_otus	Shannon	Simpson
CTL	224 ± 43.67 ^a^	20.16 ± 4.98 ^a^	224 ± 43.67 ^a^	4.62 ± 1.19	0.86 ± 0.17
ATBX	156.68 ± 28.12 ^b^	12.81 ± 1.27 ^bc^	156.68 ± 28.12 ^b^	4.22 ± 0.57	0.88 ± 0.04
EXC	147.17 ± 35.05 ^b^	13.47 ± 1.76 ^bc^	147.17 ± 35.05 ^b^	5.14 ± 0.40	0.95 ± 0.01
KGM	96.83 ± 27.78 ^c^	10.55 ± 2.13 ^c^	96.83 ± 27.78 ^c^	4.19 ± 0.84	0.89 ± 0.09
EK	183 ± 32.54 ^a^	15.75 ± 2.14 ^b^	183 ± 32.54 ^a^	4.85 ± 0.39	0.93 ± 0.03

Data are shown in average ± standard errors. Different letters indicate the significant difference among different groups for the same index, ANOVA, *p* < 0.05. N = 6.

## Data Availability

The data that support the findings of this study are not openly available due to reasons of sensitivity but are available from the corresponding author upon reasonable request. Data are located in controlled access data storage at Guangzhou Sport University.

## Supplementary Materials

The following supporting information can be downloaded at: https://www.mdpi.com/article/10.3390/nu16172942/s1, Figure S1: The daily water intake of the mice in the experiment (a) and maximum running distance (b) and Firmicutes/Bacteroidetes at different time points (c); Table S1: The chemical compounds used in this work with information from NCBI PubChem compound database and the supplier sources; Table S2: The formula of AIN93 purified diet used in the study; Table S3: Histological scoring system; Table S4: The relative abundance of top 20 taxa in feces at different levels on day 0; Table S5: The relative abundance of top 20 taxa in feces at different levels on day 14; Table S6: The relative abundance of top 20 taxa in feces at different levels on day 21; Table S7: The relative abundance of top 20 taxa in feces at different levels on day 42.
