# Supplementary material for: The Combination of Exercise and Konjac Glucomannan More Effectively Prevents Antibiotics-Induced Dysbiosis in Mice Compared with Singular Intervention
Source: Nutrients. 2024 Sep 2;16(17):2942. doi: 10.3390/nu16172942 (PMC11397520; doi:10.3390/nu16172942)
Supplement: Supplementary file 1 [file nutrients-16-02942-s001.zip › nutrients-3186231-supplementary.pdf]

# The combination of exercise and konjac glucomannan synergistically restores dysbiosis in mice induced by antibiotics compared with singular intervention

Minghan Wang [a], Xiquan Weng [a], Yan Meng [a], Jieru Lin [a], Ang-Xin Song [b], Yu-Heng Mao [a, c] *

[a] School of Exercise and Health, Guangzhou Sport University, Guangzhou 510500, China;

[b] Key Laboratory of Plant Resource Conservation and Germplasm Innovation in Mountainous Region (Ministry of Education), School of Liquor and Food Engineering, Guizhou University, Guiyang 550025, Guizhou Province, China;

[c] Guangdong Key Laboratory of Human Sports Performance Science, Guangzhou Sport University, Guangzhou 510500, China;

[*] Correspondence: maoyh@gzsport.edu.cn; Tel./Fax: +86-(020)-38024623； ORCID: 0000-0002-9602-9910.

**Table S1 The chemical compounds used in this work with information from NCBI PubChem compound database and the supplier sources**

| Compound | CAS No. | CID | MW (g/mol) | InChIKey | IUPAC name | Source |
|---|---|---|---|---|---|---|

| | | | | | | |
|---|---|---|---|---|---|---|
| Acetic acid | 64-19-7 | 176 | 60.05 | QTBSBXVTEAMEQO-UHFFFAOYSA-N | acetic acid | Aladdin® (Shanghai, China) |
| Propionic acid | 79-09-4 | 1032 | 74.08 | XBDQKXXYIPTUBI-UHFFFAOYSA-N | propanoic acid | Aladdin® (Shanghai, China) |
| *n*-butyric acid | 107-92-6 | 264 | 88.11 | FERIUCNNQQJTOY-UHFFFAOYSA-N | butynoic acid | Aladdin® (Shanghai, China) |
| *iso*-butyric acid | 79-31-2 | 6590 | 88.11 | KQNPFQTWMSNSAP-UHFFFAOYSA-N | 2-methylpropanoic acid | Aladdin® (Shanghai, China) |
| n-valeric acid | 109-52-4 | 7991 | 102.13 | NQPDZGIKBAWPEJ-UHFFFAOYSA-N | pentanoic acid | Aladdin® (Shanghai, |

| | | | | | | |
|---|---|---|---|---|---|---|
| | | | | | | China) |
| *iso*-valeric acid | 503-74-2 | 10430 | 102.13 | GWYFCOCPABKNJV-UHFFFAOYSA-N | 3-methylbutanoic acid | Aladdin® (Shanghai, China) |
| Ampicillin | 7177-48-2 | 6249 | 349.4 | RXDALBZNGVATNY-CWLIKTDRSA-N | (2S,5R,6R)-6-[[(2R)-2-amino-2-phenylacetyl]amino]-3,3-dimethyl-7-oxo-4-thia-1-azabicyclo[3.2.0]heptane-2-carboxylic acid | Macklin® (Shanghai, China) |

| | | | | | | |
|---|---|---|---|---|---|---|
| Clindamycin hydrochloride | 21462-39-5 | 16051951 | 461.4 | AUODDLQVRAJAJM-XJQDNNTCSA-N | (2S,4R)-N-[(1S,2S)-2-chloro-1-[(2R,3R,4S,5R,6R)-3,4,5-trihydroxy-6-methylsulfanyloxan-2-yl]propyl]-1-methyl-4-propylpyrrolidine-2-carboxamide;hydrochloride | Macklin® (Shanghai, China) |

| | | | | | | |
|---|---|---|---|---|---|---|
| streptomycin | 57-92-1 | 19649 | 581.6 | UCSJYZPVAKXKNQ-HZYVHMACSA-N | 2-[(1R,2R,3S,4R,5R,6S)-3-(diaminomethylideneamino)-4-[(2R,3R,4R,5S)-3-[(2S,3S,4S,5R,6S)-4,5-dihydroxy-6-(hydroxymethyl)-3-(methylamino)oxan-2-yl]oxy-4-formyl-4-hydroxy-5-methyloxolan-2- | Macklin® (Shanghai, China) |

| | | | | | | |
|---|---|---|---|---|---|---|
| | | | | | yl]oxy-2,5,6-trihydroxycyclohexyl]guanidine | |
| Paraformaldehyde | 50-00-0 | 712 | 30.026 | WSFSSNUMVMOOMR-UHFFFAOYSA-N | formaldehyde | Macklin® (Shanghai, China) |

CID: PubChem compound ID; InChIKey: International Chemical Identifier

**Table S2. The formula of AIN93 purified diet used in the study**

| Ingredient | gram | kcal |
|---|---|---|
| Casein, 30Mesh | 200 | 800 |
| L-Cystine | 3 | 12 |
| Corn Starch | 397 | 1590 |
| Maltodextrin 10 | 132 | 528 |

| | | |
|---|---|---|
| Sucrose | 100 | 400 |
| Cellulose | 50 | 0 |
| Soybean Oil | 70 | 630 |
| t-Butylhydroquinone | 0.014 | 0 |
| Mineral Mix S10022M | 35 | 0 |
| Vitamin Mix V10037 | 10 | 40 |
| Choline Bitartrate | 2.5 | 0 |
| Total | 1000 | 3850 |
| Macronutrients | | |
| Protein | 20.0 (gram%) | 20.3 (Kcal%) |
| Carbohydrate | 64.0 (gram%) | 63.9 (Kcal%) |
| Fat | 7.0 (gram%) | 15.8 (Kcal%) |
| Total | | 100.0 (Kcal%) |
| kcal/gm | 3.9 | |

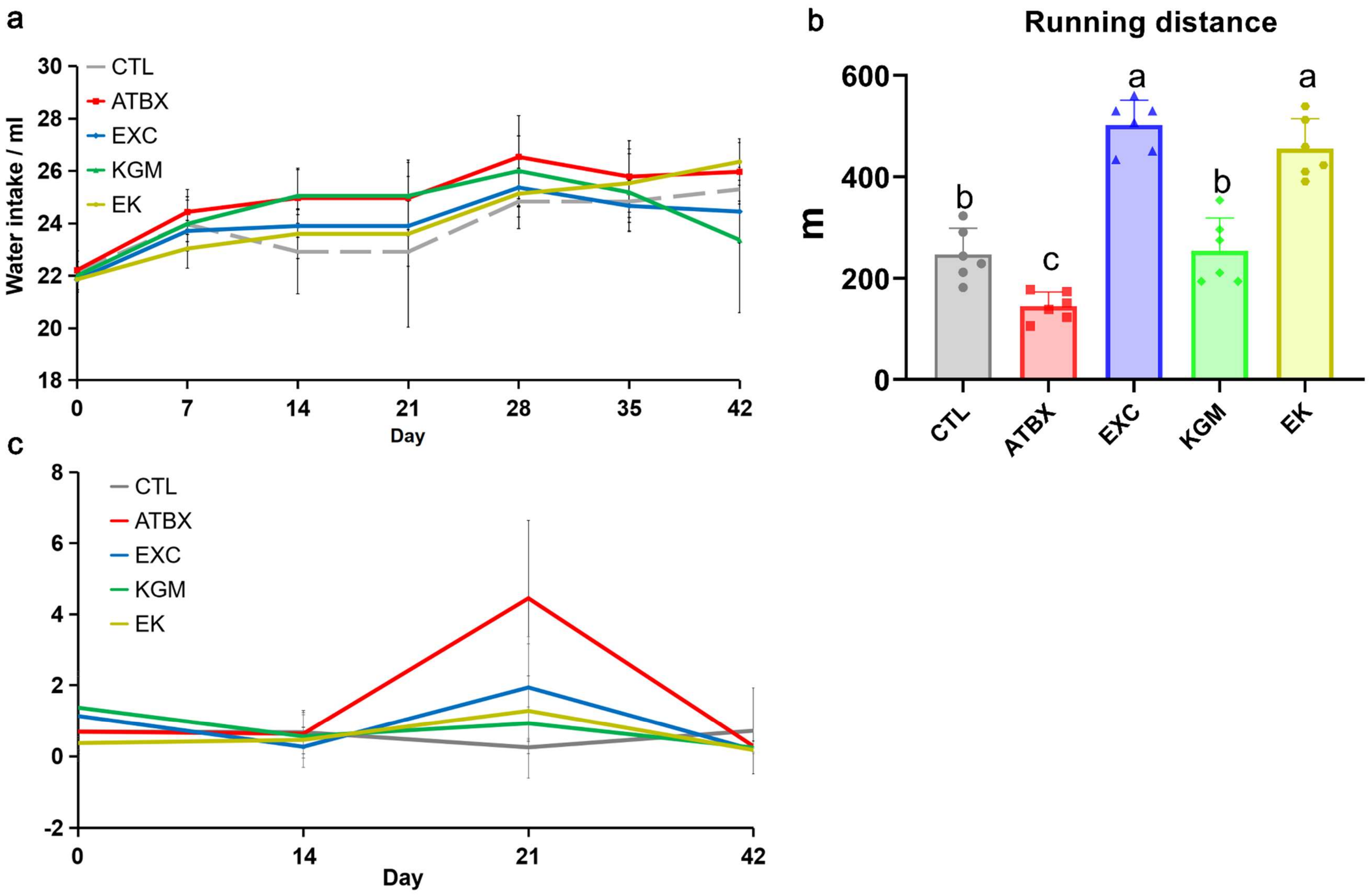
a
Water intake / ml
CTL
ATBX
EXC
KGM
EK
Day
b
Running distance
m
b
c
a
b
a
CTL
ATBX
EXC
KGM
EK
c
CTL
ATBX
EXC
KGM
EK
Day

Fig S1 The daily water intake of the mice in the experiment (a) and maximum running distance (b) and Firmicutes/Bacteroidetes at different time points (c). Average of six samples, error bars represent standard deviation at N = 6. (c)Different letters indicate the significant difference among different groups for the same index, ANOVA with Bonferroni or Tamhane T2 post-hoc test, $p < 0.05$. CTL: control; ATBX: antibiotic; EXC: exercise; KGM: the native KGM; EK: combination of exercise and KGM.

**Table S3 Histological scoring system**

| Histological score | Degree of inflammation | Infiltration of inflammatory | Degree of damage to the crypt | Crypt abscesses | Degree of submucosal edema | Reduction of goblet cells | Degree of epithelial hyperplasia |
|---|---|---|---|---|---|---|---|
| 0 | Normal | Normal | Normal | Normal | Normal | Normal | Normal |
| 1 | Mucosa | Unifocal | Basal 1/3 of crypt | Unifocal | Unifocal | Unifocal | Unifocal |

| | | | | | | | |
|---|---|---|---|---|---|---|---|
| 2 | Submucosa | Multifocal | Basal 2/3 of crypt | Multifocal | Multifocal | Multifocal | Multifocal |
| 3 | Muscular | Suffuse | Suffuse | | Suffuse | Suffuse | Suffuse |
| 4 | Serous | | Damage to the crypt and ulceration | | | | |

**Table S4 The relative abundance of top 20 taxa in feces at different levels on day 0**

| Day 0 | CTL | ATBX | EXC | KGM | EK |
|---|---|---|---|---|---|
| **Phylum** | | | | | |
| Bacteroidetes | 60.74 ± 19.58 ab | 59.45 ± 17.95 ab | 55.57 ± 22.94 ab | 45.81 ± 19.73 b | 69.65 ± 9.39 a |

| | | | | | |
|---|---|---|---|---|---|
| Firmicutes | 32.45 ± 18.47 ab | 33.62 ± 16.93 ab | 39.71 ± 24.97 ab | 48.70 ± 18.02 a | 24.45 ± 8.96 b |
| Tenericutes | 4.96 ± 3.97 a | 1.15 ± 0.79 b | 3.16 ± 3.40 ab | 2.46 ± 2.56 ab | 3.58 ± 3.36 ab |
| Proteobacteria | 0.55 ± 0.51 b | 2.66 ± 1.28 a | 0.61 ± 0.50 b | 2.08 ± 4.18 b | 1.66 ± 1.63 ab |
| Actinobacteria | 0.60 ± 0.56 | 2.80 ± 5.21 | 0.40 ± 0.41 | 0.53 ± 0.73 | 0.26 ± 0.15 |
| TM7 | 0.58 ± 0.54 | 0.16 ± 0.17 | 0.55 ± 0.60 | 0.34 ± 0.14 | 0.19 ± 0.15 |
| Deferribacteres | 0.11 ± 0.22 | 0.12 ± 0.14 | 0.00 ± 0.01 | 0.08 ± 0.11 | 0.09 ± 0.11 |
| Crenarchaeota | 0.00 ± 0.00 b | 0.01 ± 0.02 ab | 0.00 ± 0.00 b | 0.00 ± 0.00 b | 0.05 ± 0.08 a |
| Fusobacteria | 0.00 ± 0.00 b | 0.00 ± 0.01 b | 0.00 ± 0.00 b | 0.00 ± 0.00 b | 0.02 ± 0.02 a |
| unclassified | 0.00 ± 0.00 | 0.01 ± 0.01 | 0.00 ± 0.00 | 0.00 ± 0.00 | 0.01 ± 0.01 |
| Acidobacteria | 0.00 ± 0.00 | 0.00 ± 0.00 | 0.00 ± 0.00 | 0.00 ± 0.00 | 0.01 ± 0.01 |
| Cyanobacteria | 0.00 ± 0.00 | 0.00 ± 0.00 | 0.00 ± 0.00 | 0.00 ± 0.00 | 0.01 ± 0.01 |

| | | | | | |
|---|---|---|---|---|---|
| Chloroflexi | 0.00 ± 0.00 | 0.00 ± 0.00 | 0.00 ± 0.00 | 0.00 ± 0.00 | 0.00 ± 0.00 |
| Verrucomicrobia | 0.00 ± 0.00 | 0.00 ± 0.00 | 0.00 ± 0.00 | 0.00 ± 0.00 | 0.00 ± 0.01 |
| Gemmatimonadetes | 0.00 ± 0.00 b | 0.00 ± 0.00 a | 0.00 ± 0.00 b | 0.00 ± 0.00 b | 0.00 ± 0.00 ab |
| Euryarchaeota | 0.00 ± 0.00 | 0.00 ± 0.00 | 0.00 ± 0.00 | 0.00 ± 0.01 | 0.00 ± 0.00 |
| Spirochaetes | 0.00 ± 0.00 b | 0.00 ± 0.00 ab | 0.00 ± 0.00 b | 0.00 ± 0.00 b | 0.00 ± 0.00 a |
| Nitrospirae | 0.00 ± 0.00 | 0.00 ± 0.00 | 0.00 ± 0.00 | 0.00 ± 0.00 | 0.00 ± 0.00 |
| Planctomycetes | 0.00 ± 0.00 | 0.00 ± 0.00 | 0.00 ± 0.00 | 0.00 ± 0.00 | 0.00 ± 0.00 |
| SAR406 | 0.00 ± 0.00 | 0.00 ± 0.00 | 0.00 ± 0.00 | 0.00 ± 0.00 | 0.00 ± 0.00 |
| Other | 0.00 ± 0.00 b | 0.00 ± 0.00 ab | 0.00 ± 0.00 b | 0.00 ± 0.00 b | 0.00 ± 0.00 a |
| **Family** | | | | | |
| S24_7 | 59.22 ± 19.16 ab | 57.57 ± 17.40 ab | 53.84 ± 22.27 ab | 44.01 ± 18.70 b | 66.82 ± 10.58 a |
| Lactobacillaceae | 26.94 ± 16.14 | 21.83 ± 15.77 | 34.54 ± 28.33 | 37.17 ± | 12.08 ± 9.58 |

| | | | | | |
|---|---|---|---|---|---|
| | | | | 28.02 | |
| unclassified | 6.70 ± 4.16 | 4.27 ± 1.86 | 5.69 ± 5.25 | 5.87 ± 3.64 | 7.49 ± 3.91 |
| Lachnospiraceae | 2.85 ± 2.45 | 5.16 ± 3.22 | 1.96 ± 2.32 | 6.57 ± 8.12 | 5.84 ± 4.72 |
| Ruminococcaceae | 0.37 ± 0.20 b | 1.97 ± 1.23 a | 0.39 ± 0.39 b | 0.99 ± 1.48 ab | 1.93 ± 1.48 b |
| Bacteroidaceae | 0.64 ± 0.51 | 0.82 ± 1.02 | 1.34 ± 1.04 | 0.99 ± 0.72 | 1.63 ± 1.70 |
| Enterobacteriaceae | 0.37 ± 0.53 | 2.09 ± 0.93 | 0.43 ± 0.52 | 1.85 ± 4.16 | 0.27 ± 0.16 |
| Rikenellaceae | 0.61 ± 0.38 | 1.04 ± 1.21 | 0.38 ± 0.28 | 0.48 ± 0.49 | 0.92 ± 0.64 |
| Coriobacteriaceae | 0.60 ± 0.56 | 0.58 ± 0.53 | 0.40 ± 0.41 | 0.52 ± 0.73 | 0.22 ± 0.19 |
| Micrococcaceae | 0.00 ± 0.00 | 2.11 ± 5.17 | 0.00 ± 0.00 | 0.00 ± 0.00 | 0.00 ± 0.01 |
| F16 | 0.58 ± 0.54 | 0.16 ± 0.17 | 0.55 ± 0.60 | 0.34 ± 0.14 | 0.19 ± 0.15 |
| Clostridiaceae | 0.17 ± 0.09 | 0.49 ± 0.35 | 0.12 ± 0.21 | 0.21 ± 0.20 | 0.13 ± 0.09 |
| Erysipelotrichaceae | 0.06 ± 0.05 | 0.32 ± 0.42 | 0.15 ± 0.26 | 0.03 ± 0.03 | 0.38 ± 0.54 |
| Porphyromonadaceae | 0.27 ± 0.25 b | 0.01 ± 0.01 c | 0.00 ± 0.00 c | 0.32 ± 0.77 a | 0.23 ± 0.26 |

| | | | | | |
|---|---|---|---|---|---|
| | | | | | bc |
| Turicibacteraceae | 0.29 ± 0.21 a | 0.23 ± 0.23 ab | 0.02 ± 0.02 b | 0.27 ± 0.34 ab | 0.01 ± 0.02 b |
| Brucellaceae | 0.02 ± 0.03 b | 0.13 ± 0.16 ab | 0.02 ± 0.05 b | 0.04 ± 0.05 b | 0.55 ± 0.70 a |
| Pseudomonadaceae | 0.02 ± 0.02 b | 0.16 ± 0.19 ab | 0.03 ± 0.04 b | 0.07 ± 0.07 ab | 0.44 ± 0.45 a |
| Deferribacteraceae | 0.11 ± 0.22 | 0.12 ± 0.14 | 0.00 ± 0.01 | 0.08 ± 0.11 | 0.09 ± 0.11 |
| Alcaligenaceae | 0.11 ± 0.09 | 0.08 ± 0.11 | 0.06 ± 0.06 | 0.03 ± 0.02 | 0.03 ± 0.02 |
| Planococcaceae | 0.00 ± 0.00 | 0.28 ± 0.68 | 0.00 ± 0.00 | 0.00 ± 0.00 | 0.00 ± 0.00 |
| Other | 0.07 ± 0.04 | 0.57 ± 0.47 | 0.09 ± 0.06 | 0.18 ± 0.14 | 0.75 ± 0.61 |
| **Genus** | | | | | |
| unclassified | 70.08 ± 16.55 | 70.89 ± 15.56 | 62.62 ± 27.04 | 58.52 ± 25.08 | 80.93 ± 7.40 |
| *Lactobacillus* | 26.94 ± 16.14 | 21.83 ± 15.78 | 34.54 ± 28.33 | 37.17 ± | 12.08 ± 9.58 |

| | | | | | |
|---|---|---|---|---|---|
| | | | | 28.02 | |
| *Bacteroides* | 0.64 ± 0.51 | 0.82 ± 1.02 | 1.34 ± 1.04 | 0.99 ± 0.72 | 1.63 ± 1.70 |
| *Anaerostipes* | 0.27 ± 0.18 | 0.47 ± 0.24 | 0.25 ± 0.21 | 0.37 ± 0.26 | 1.10 ± 1.50 |
| *Kocuria* | 0.00 ± 0.00 | 2.09 ± 5.13 | 0.00 ± 0.00 | 0.00 ± 0.00 | 0.00 ± 0.00 |
| *Adlercreutzia* | 0.56 ± 0.55 | 0.49 ± 0.45 | 0.32 ± 0.33 | 0.47 ± 0.74 | 0.20 ± 0.17 |
| *Oscillospira* | 0.11 ± 0.10 | 0.58 ± 0.50 | 0.09 ± 0.08 | 0.35 ± 0.48 | 0.57 ± 0.55 |
| *Ruminococcaceae_Ruminococcus* | 0.03 ± 0.03 b | 0.20 ± 0.19 a | 0.03 ± 0.03 b | 0.35 ± 0.75 ab | 0.65 ± 0.89 a |
| *Coprococcus* | 0.09 ± 0.07 | 0.45 ± 0.49 | 0.10 ± 0.15 | 0.26 ± 0.42 | 0.36 ± 0.41 |
| *Ruminococcus* | 0.11 ± 0.09 | 0.25 ± 0.21 | 0.19 ± 0.18 | 0.22 ± 0.35 | 0.33 ± 0.26 |
| *Clostridium* | 0.17 ± 0.09 | 0.47 ± 0.32 | 0.12 ± 0.21 | 0.20 ± 0.20 | 0.13 ± 0.09 |
| *Parabacteroides* | 0.27 ± 0.25b | 0.01 ± 0.01c | 0.00 ± 0.00c | 0.32 ± 0.77a | 0.23 ± 0.26bc |
| *Turicibacter* | 0.29 ± 0.21 a | 0.23 ± 0.23 ab | 0.02 ± 0.02 b | 0.27 ± 0.34 | 0.01 ± 0.02 b |

| | | | | | |
|---|---|---|---|---|---|
| | | | | ab | |
| *Roseburia* | 0.11 ± 0.19 | 0.17 ± 0.20 | 0.09 ± 0.17 | 0.12 ± 0.25 | 0.28 ± 0.31 |
| *Pseudomonas* | 0.02 ± 0.02 b | 0.16 ± 0.19 ab | 0.03 ± 0.04 b | 0.07 ± 0.07 ab | 0.43 ± 0.45 a |
| *Mucispirillum* | 0.11 ± 0.22 | 0.12 ± 0.14 | 0.00 ± 0.01 | 0.08 ± 0.11 | 0.09 ± 0.11 |
| *Sutterella* | 0.10 ± 0.09 | 0.07 ± 0.11 | 0.05 ± 0.06 | 0.02 ± 0.01 | 0.02 ± 0.02 |
| *Coprobacillus* | 0.01 ± 0.02 | 0.05 ± 0.04 | 0.11 ± 0.22 | 0.02 ± 0.03 | 0.06 ± 0.10 |
| *Plesiomonas* | 0.01 ± 0.01 b | 0.07 ± 0.06 ab | 0.01 ± 0.01 b | 0.03 ± 0.04 b | 0.11 ± 0.11 a |
| *Ralstonia* | 0.00 ± 0.00 b | 0.04 ± 0.04 ab | 0.00 ± 0.01 b | 0.01 ± 0.02 b | 0.10 ± 0.08 a |
| Other | 0.07 ± 0.04 | 0.55 ± 0.51 | 0.09 ± 0.05 | 0.17 ± 0.13 | 0.68 ± 0.60 |

Data are shown in average ± standard deviation. Different letters indicate the significant difference among different groups for the same index, ANOVA with LSD or Tamhane T2 post-hoc test, $p < 0.05$. N = 6. CTL: control; ATBX: antibiotic; EXC: exercise; KGM: the native KGM; EK: combination of exercise and KGM.

**Table S5 The relative abundance of top 20 taxa in feces at different levels on day 14**

| Day 14 | CTL | ATBX | EXC | KGM | EK |
|---|---|---|---|---|---|
| **Phylum** | | | | | |
| Bacteroidetes | 52.70 ± 20.39 b | 56.75 ± 9.44 b | 77.64 ± 3.45 a | 50.19 ± 22.27 b | 73.57 ± 26.44 a |
| Firmicutes | 25.95 ± 13.71 | 34.44 ± 5.69 | 20.58 ± 3.57 | 28.81 ± 21.51 | 16.92 ± 12.23 |
| Verrucomicrobia | 13.53 ± 11.20 a | 7.33 ± 6.14 a | 0.31 ± 0.39 b | 16.67 ± 20.60 a | 2.06 ± 1.81 ab |
| Proteobacteria | 3.40 ± 2.44 a | 0.62 ± 0.18 b | 0.99 ± 0.38 ab | 0.33 ± 0.37 b | 0.53 ± 0.50 b |
| Actinobacteria | 4.24 ± 6.22 a | 0.24 ± 0.20 b | 0.17 ± 0.11 b | 0.20 ± 0.20 b | 1.66 ± 3.34 ab |
| Tenericutes | 0.14 ± 0.21 | 0.18 ± 0.21 | 0.24 ± 0.15 | 3.55 ± 8.16 | 0.24 ± 0.23 |
| Deferribacteres | 0.01 ± 0.02 b | 0.39 ± 0.16 a | 0.04 ± 0.04 b | 0.09 ± 0.09 b | 0.14 ± 0.21 b |

| | | | | | |
|---|---|---|---|---|---|
| Cyanobacteria | 0.03 ± 0.04 | 0.02 ± 0.04 | 0.02 ± 0.03 | 0.05 ± 0.05 | 3.69 ± 8.98 |
| TM7 | 0.01 ± 0.01 | 0.03 ± 0.07 | 0.02 ± 0.02 | 0.05 ± 0.06 | 0.06 ± 0.12 |
| Acidobacteria | 0.00 ± 0.00 | 0.00 ± 0.00 | 0.00 ± 0.04 | 0.02 ± 0.41 | 0.17 ± 0.18 |
| unclassified | 0.00 ± 0.00 | 0.00 ± 0.00 | 0.00 ± 0.00 | 0.01 ± 0.02 | 0.02 ± 0.03 |
| Gemmatimonadetes | 0.00 ± 0.00 | 0.00 ± 0.00 | 0.00 ± 0.00 | 0.01 ± 0.02 | 0.00 ± 0.01 |
| Planctomycetes | 0.00 ± 0.00 | 0.00 ± 0.00 | 0.00 ± 0.00 | 0.00 ± 0.01 | 0.07 ± 0.17 |
| Chloroflexi | 0.00 ± 0.00 | 0.00 ± 0.00 | 0.00 ± 0.00 | 0.00 ± 0.01 | 0.02 ± 0.04 |
| Euryarchaeota | 0.00 ± 0.00 | 0.00 ± 0.00 | 0.00 ± 0.00 | 0.00 ± 0.00 | 0.02 ± 0.06 |
| Nitrospirae | 0.00 ± 0.00 | 0.00 ± 0.00 | 0.00 ± 0.00 | 0.00 ± 0.00 | 0.03 ± 0.07 |
| Chlorobi | 0.00 ± 0.00 | 0.00 ± 0.00 | 0.00 ± 0.00 | 0.00 ± 0.00 | 0.36 ± 0.88 |
| Crenarchaeota | 0.00 ± 0.00 | 0.00 ± 0.00 | 0.00 ± 0.00 | 0.00 ± 0.00 | 0.08 ± 0.21 |
| Armatimonadetes | 0.00 ± 0.00 | 0.00 ± 0.00 | 0.00 ± 0.00 | 0.00 ± 0.00 | 0.06 ± 0.15 |
| Fusobacteria | 0.00 ± 0.00 | 0.00 ± 0.00 | 0.00 ± 0.00 | 0.00 ± 0.00 | 0.01 ± 0.02 |
| Other | 0.00 ± 0.00 | 0.00 ± 0.00 | 0.00 ± 0.00 | 0.00 ± 0.00 | 0.29 ± 0.72 |

| **Family** | | | | | |
|---|---|---|---|---|---|
| S24_7 | 35.12 ± 15.21 ab | 45.82 ± 5.60 b | 62.13 ± 3.87 a | 41.36 ± 18.41 ab | 53.11 ± 17.83 ab |
| Erysipelotrichaceae | 10.45 ± 11.12 | 18.73 ± 9.90 | 6.10 ± 2.77 | 9.97 ± 12.27 | 7.90 ± 16.33 |
| unclassified | 3.65 ± 3.85 | 10.61 ± 7.89 | 7.59 ± 3.15 | 16.29 ± 25.47 | 6.29 ± 3.86 |
| Verrucomicrobiaceae | 13.53 ± 11.20 a | 7.33 ± 6.14 a | 0.31 ± 0.39 b | 16.67 ± 20.60 a | 2.01 ± 1.85 ab |
| Paraprevotellaceae | 4.60 ± 4.84 b | 7.40 ± 5.79 ab | 8.42 ± 6.26 ab | 0.71 ± 1.73 b | 17.77 ± 11.24 a |
| Bacteroidaceae | 4.84 ± 2.32 | 1.15 ± 0.44 | 5.24 ± 2.93 | 6.49 ± 4.44 | 3.00 ± 2.35 |
| Lactobacillaceae | 7.90 ± 6.94 | 1.30 ± 1.23 | 4.04 ± 4.00 | 1.78 ± 1.37 | 0.27 ± 0.25 |
| Lachnospiraceae | 2.10 ± 0.97 | 1.55 ± 0.99 | 1.79 ± 1.44 | 2.98 ± 4.86 | 4.65 ± 8.54 |
| Ruminococcaceae | 1.21 ± 1.01 | 2.36 ± 0.77 | 1.23 ± 0.85 | 1.31 ± 0.76 | 1.75 ± 1.43 |
| Rikenellaceae | 3.30 ± 2.80 | 1.01 ± 0.45 | 1.65 ± 1.70 | 0.43 ± 0.22 | 0.57 ± 0.37 |

| | | | | | |
|---|---|---|---|---|---|
| Porphyromonadaceae | 4.14 ± 4.28 | 0.75 ± 0.59 | 0.01 ± 0.01 | 1.19 ± 0.91 | 0.04 ± 0.03 |
| Bifidobacteriaceae | 4.10 ± 6.22 a | 0.14 ± 0.12 ab | 0.01 ± 0.01 b | 0.04 ± 0.04 b | 0.02 ± 0.04 b |
| Helicobacteraceae | 2.48 ± 2.72 a | 0.00 ± 0.00 b | 0.27 ± 0.29 a | 0.00 ± 0.00 b | 0.07 ± 0.17 b |
| Alcaligenaceae | 0.70 ± 0.82 a | 0.54 ± 0.16 a | 0.64 ± 0.41 a | 0.12 ± 0.07 b | 0.45 ± 0.51 ab |
| Prevotellaceae | 0.69 ± 0.88 a | 0.62 ± 0.89 a | 0.18 ± 0.10 a | 0.00 ± 0.00 b | 0.71 ± 0.83 a |
| Coriobacteriaceae | 0.14 ± 0.08 | 0.10 ± 0.09 | 0.16 ± 0.10 | 0.13 ± 0.17 | 0.26 ± 0.21 |
| Deferribacteraceae | 0.01 ± 0.02 b | 0.39 ± 0.16 a | 0.04 ± 0.04 b | 0.09 ± 0.09 b | 0.50 ± 0.84 ab |
| Peptostreptococcaceae | 0.29 ± 0.45 a | 0.00 ± 0.00 b | 0.00 ± 0.00 b | 0.00 ± 0.00 b | 0.08 ± 0.21 b |
| Enterococcaceae | 0.24 ± 0.25 a | 0.01 ± 0.01 b | 0.00 ± 0.00 b | 0.00 ± 0.00 b | 0.06 ± 0.15 b |
| Enterobacteriaceae | 0.16 ± 0.21 a | 0.00 ± 0.00 b | 0.04 ± 0.02 | 0.02 ± 0.02 b | 0.02 ± 0.03 b |

| | | | | | |
|---|---|---|---|---|---|
| | | | b | | |
| Other | 0.34 ± 0.11 | 0.21 ± 0.10 | 0.15 ± 0.08 | 0.43 ± 0.41 | 0.47 ± 0.64 |
| **Genus** | | | | | |
| unclassified | 43.83 ± 16.33 b | 59.31 ± 7.91 b | 72.45 ± 3.39 a | 61.13 ± 18.98 ab | 60.94 ± 21.21 ab |
| *Allobaculum* | 7.59 ± 8.89 | 18.68 ± 9.89 | 6.06 ± 2.77 | 9.83 ± 12.23 | 7.87 ± 16.34 |
| *Akkermansia* | 13.53 ± 11.20 a | 7.33 ± 6.14 b | 0.31 ± 0.39 c | 16.67 ± 20.61 a | 2.06 ± 1.81 bc |
| *Prevotella* | 4.60 ± 4.84 ab | 7.40 ± 5.79 a | 8.42 ± 6.26 a | 0.71 ± 1.73 b | 16.43 ± 12.92 a |
| *Bacteroides* | 4.84 ± 2.32 | 1.15 ± 0.44 | 5.24 ± 2.93 | 6.49 ± 4.44 | 4.40 ± 2.72 |
| *Lactobacillus* | 7.90 ± 6.94 | 1.30 ± 1.23 | 4.04 ± 4.00 | 1.78 ± 1.37 | 0.27 ± 0.24 |
| *Parabacteroides* | 4.14 ± 4.28 | 0.75 ± 0.59 | 0.01 ± 0.01 | 1.19 ± 0.91 | 0.03 ± 0.03 |
| *Bifidobacterium* | 4.10 ± 6.22 a | 0.14 ± 0.12 b | 0.01 ± 0.01 | 0.04 ± 0.04 b | 3.69 ± 8.98 b |

| | | | | | |
|---|---|---|---|---|---|
| | | | b | | |
| *Oscillospira* | 0.81 ± 0.89 | 0.84 ± 0.32 | 0.72 ± 0.56 | 0.33 ± 0.20 | 0.69 ± 0.62 |
| *Helicobacter* | 2.48 ± 2.72 a | 0.00 ± 0.00 b | 0.27 ± 0.29 a | 0.00 ± 0.00 b | 0.17 ± 0.41 b |
| *Ruminococcaceae_Ruminococcus* | 0.21 ± 0.10 | 0.81 ± 0.33 | 0.29 ± 0.19 | 0.59 ± 0.42 | 0.76 ± 0.65 |
| *Sutterella* | 0.69 ± 0.82 a | 0.54 ± 0.16 a | 0.64 ± 0.41 a | 0.12 ± 0.07 b | 0.44 ± 0.52 ab |
| *Coprococcus* | 0.68 ± 0.91 | 0.34 ± 0.27 | 0.86 ± 1.00 | 0.27 ± 0.32 | 0.23 ± 0.19 |
| *Prevotellaceae_Prevotella* | 0.69 ± 0.88 a | 0.62 ± 0.89 a | 0.18 ± 0.10 a | 0.00 ± 0.00 b | 0.70 ± 0.84 a |
| *Eubacterium* | 1.26 ± 2.26 a | 0.01 ± 0.01 b | 0.00 ± 0.00 b | 0.00 ± 0.00 b | 0.02 ± 0.06 b |
| *Erysipelotrichaceae_Clostridium* | 0.74 ± 1.35 a | 0.02 ± 0.02 b | 0.02 ± 0.03 b | 0.08 ± 0.03 ab | 0.05 ± 0.06 b |
| *Mucispirillum* | 0.01 ± 0.02 b | 0.39 ± 0.16 a | 0.04 ± 0.04 b | 0.09 ± 0.09 b | 0.50 ± 0.84 ab |
| *Ruminococcus* | 0.18 ± 0.22 | 0.13 ± 0.05 | 0.09 ± 0.06 | 0.20 ± 0.38 | 0.12 ± 0.19 |

| | | | | | |
|---|---|---|---|---|---|
| *Rikenella* | 0.54 ± 0.67 a | 0.00 ± 0.00 b | 0.00 ± 0.00 b | 0.00 ± 0.00 b | 0.06 ± 0.15 b |
| *Adlercreutzia* | 0.14 ± 0.07 | 0.07 ± 0.06 | 0.14 ± 0.09 | 0.08 ± 0.05 | 0.07 ± 0.03 |
| Other | 1.05 ± 0.72 a | 0.19 ± 0.13 b | 0.22 ± 0.21 b | 0.41 ± 0.36 b | 0.50 ± 0.64 b |

Data are shown in average ± standard deviation. Different letters indicate the significant difference among different groups for the same index, ANOVA with LSD or Tamhane T2 post-hoc test, $p < 0.05$. N = 6. CTL: control; ATBX: antibiotic; EXC: exercise; KGM: the native KGM; EK: combination of exercise and KGM.

**Table S6 The relative abundance of top 20 taxa in feces at different levels on day 21**

| Day 21 | CTL | ATBX | EXC | KGM | EK |
|---|---|---|---|---|---|
| **Phylum** | | | | | |
| Proteobacteria | 0.54 ± 0.37 b | 92.72 ± 5.30 | 96.77 ± 2.17 | 70.50 ± 11.98 | 92.37 ± 10.95 |

| | | | | | |
|---|---|---|---|---|---|
| | | ab | a | b | a |
| Bacteroidetes | 72.83 ± 7.16 a | 1.54 ± 1.40 b | 1.17 ± 1.04 b | 15.85 ± 8.45 a | 5.77 ± 8.91 b |
| Firmicutes | 17.31 ± 9.47 a | 5.08 ± 3.32 b | 1.78 ± 1.17 b | 11.98 ± 3.49 ab | 1.36 ± 1.24 b |
| Verrucomicrobia | 8.17 ± 4.38 a | 0.05 ± 0.07 b | 0.06 ± 0.06 b | 0.87 ± 0.38 ab | 0.00 ± 0.01 b |
| Actinobacteria | 0.99 ± 0.63 a | 0.18 ± 0.15 b | 0.11 ± 0.10 b | 0.54 ± 0.20 a | 0.19 ± 0.39 b |
| unclassified | 0.00 ± 0.00 | 0.30 ± 0.62 | 0.05 ± 0.05 | 0.09 ± 0.08 | 0.13 ± 0.30 |
| Tenericutes | 0.13 ± 0.12 a | 0.01 ± 0.01 b | 0.00 ± 0.00 b | 0.02 ± 0.01 b | 0.13 ± 0.30 b |
| Euryarchaeota | 0.00 ± 0.00 b | 0.07 ± 0.11 a | 0.03 ± 0.02 a | 0.04 ± 0.02 a | 0.04 ± 0.05 a |
| Fusobacteria | 0.00 ± 0.00 b | 0.01 ± 0.02 ab | 0.00 ± 0.00 b | 0.02 ± 0.02 a | 0.00 ± 0.00 b |
| Cyanobacteria | 0.01 ± 0.01 | 0.01 ± 0.01 | 0.00 ± 0.00 | 0.01 ± 0.01 | 0.00 ± 0.00 |

| | | | | | |
|---|---|---|---|---|---|
| Acidobacteria | 0.00 ± 0.00 b | 0.01 ± 0.01 ab | 0.00 ± 0.01 ab | 0.02 ± 0.02 a | 0.00 ± 0.00 ab |
| Deferribacteres | 0.01 ± 0.01 | 0.01 ± 0.01 | 0.00 ± 0.00 | 0.01 ± 0.01 | 0.00 ± 0.00 |
| Gemmatimonadetes | 0.00 ± 0.00 | 0.01 ± 0.01 | 0.00 ± 0.01 | 0.01 ± 0.01 | 0.00 ± 0.00 |
| TM7 | 0.01 ± 0.01a | 0.00 ± 0.00b | 0.00 ± 0.01b | 0.00 ± 0.00ab | 0.00 ± 0.00b |
| Chloroflexi | 0.00 ± 0.00 | 0.00 ± 0.01 | 0.00 ± 0.00 | 0.01 ± 0.01 | 0.00 ± 0.00 |
| Crenarchaeota | 0.00 ± 0.00 | 0.00 ± 0.00 | 0.00 ± 0.00 | 0.01 ± 0.01 | 0.00 ± 0.01 |
| Spirochaetes | 0.00 ± 0.00 | 0.00 ± 0.00 | 0.00 ± 0.00· | 0.01 ± 0.02 | 0.00 ± 0.00 |
| Nitrospirae | 0.00 ± 0.00 b | 0.00 ± 0.00 b | 0.00 ± 0.00 b | 0.00 ± 0.00 a | 0.00 ± 0.00 b |
| Planctomycetes | 0.00 ± 0.00 | 0.00 ± 0.00 | 0.00 ± 0.00 | 0.00 ± 0.00 | 0.00 ± 0.00 |
| Thermi | 0.00 ± 0.00 | 0.00 ± 0.01 | 0.00 ± 0.00 | 0.00 ± 0.00 | 0.00 ± 0.00 |
| Other | 0.00 ± 0.00 ab | 0.00 ± 0.00 a | 0.00 ± 0.00 b | 0.01 ± 0.01 a | 0.00 ± 0.00 b |
| **Family** | | | | | |

| | | | | | |
|---|---|---|---|---|---|
| Burkholderiaceae | 0.00 ± 0.00 b | 64.21 ± 35.02 ab | 81.54 ± 11.62 a | 21.96 ± 9.89 b | 41.69 ± 31.52 ab |
| Enterobacteriaceae | 0.04 ± 0.05 b | 18.02 ± 19.95 ab | 7.44 ± 6.85 b | 16.48 ± 5.75 ab | 38.49 ± 26.43 a |
| S24_7 | 57.04 ± 8.21 a | 0.41 ± 0.44 b | 0.53 ± 0.62 b | 11.07 ± 6.69 a | 0.19 ± 0.28 b |
| Halomonadaceae | 0.01 ± 0.00 b | 7.20 ± 6.79 b | 6.16 ± 3.72 b | 25.95 ± 4.84 a | 1.77 ± 1.17 b |
| Bacteroidaceae | 2.84 ± 3.41 a | 0.68 ± 0.62 ab | 0.30 ± 0.16 b | 1.93 ± 0.86 a | 5.44 ± 8.59 a |
| Erysipelotrichaceae | 8.64 ± 5.42 a | 0.98 ± 1.67 b | 0.12 ± 0.09 b | 1.21 ± 0.47 ab | 0.07 ± 0.13 b |
| Verrucomicrobiaceae | 8.17 ± 4.38 a | 0.05 ± 0.07 b | 0.06 ± 0.06 b | 0.86 ± 0.38 ab | 0.00 ± 0.01 b |

| | | | | | |
|---|---|---|---|---|---|
| Moraxellaceae | 0.00 ± 0.00 b | 0.52 ± 0.63 ab | 0.20 ± 0.18 b | 0.71 ± 0.58 ab | 7.47 ± 9.93 a |
| unclassified | 5.12 ± 6.00 a | 0.58 ± 0.73 b | 0.16 ± 0.14 b | 0.88 ± 0.48 ab | 0.26 ± 0.44 b |
| Bacillaceae | 0.00 ± 0.00 | 2.15 ± 1.92 | 0.74 ± 0.47 | 2.90 ± 1.79 | 0.38 ± 0.32 |
| Paraprevotellaceae | 4.78 ± 4.43 a | 0.12 ± 0.13 b | 0.12 ± 0.15 b | 0.71 ± 0.44 ab | 0.01 ± 0.03 b |
| Porphyromonadaceae | 3.78 ± 2.91 a | 0.05 ± 0.05 b | 0.05 ± 0.04 b | 0.85 ± 0.53 ab | 0.00 ± 0.01 b |
| Alcaligenaceae | 0.37 ± 0.35 b | 1.30 ± 1.60 ab | 0.55 ± 0.27 b | 1.69 ± 0.25 a | 0.51 ± 0.22 b |
| Staphylococcaceae | 0.00 ± 0.00 b | 0.11 ± 0.14 b | 0.06 ± 0.05 b | 3.96 ± 2.67 a | 0.24 ± 0.15 ab |
| Lactobacillaceae | 2.45 ± 1.77 a | 0.35 ± 0.53 b | 0.11 ± 0.10 b | 0.59 ± 0.26 | 0.11 ± 0.14 b |

| | | | | | |
|---|---|---|---|---|---|
| | | | | ab | |
| Prevotellaceae | 2.83 ± 2.42 a | 0.16 ± 0.11 b | 0.09 ± 0.07 b | 0.46 ± 0.37 ab | 0.03 ± 0.07 b |
| Pseudomonadaceae | 0.00 ± 0.00 b | 0.28 ± 0.25 a | 0.10 ± 0.08 ab | 0.78 ± 0.91 a | 1.52 ± 2.55 a |
| Lachnospiraceae | 0.50 ± 0.48 ab | 0.32 ± 0.21 ab | 0.20 ± 0.19 b | 1.09 ± 0.82 a | 0.18 ± 0.25 b |
| Ruminococcaceae | 0.56 ± 0.56 ab | 0.42 ± 0.27 ab | 0.20 ± 0.17 b | 0.83 ± 0.55 a | 0.06 ± 0.15 b |
| Rikenellaceae | 1.56 ± 2.09 a | 0.03 ± 0.03 b | 0.04 ± 0.02 b | 0.43 ± 0.26 ab | 0.01 ± 0.02 b |
| Other | 1.32 ± 0.77 b | 2.06 ± 2.31 b | 1.23 ± 0.66 b | 4.67 ± 0.64 a | 1.56 ± 2.32 b |
| **Genus** | | | | | |

| | | | | | |
|---|---|---|---|---|---|
| unclassified | 65.37 ± 6.99 a | 19.32 ± 21.18 b | 8.39 ± 7.40 c | 29.56 ± 9.17 b | 38.97 ± 25.77 ab |
| *Burkholderia* | 0.00 ± 0.00 b | 64.20 ± 35.02 ab | 81.51 ± 11.61 a | 0.19 ± 0.09 b | 0.00 ± 0.00 b |
| *Pandoraea* | 0.00 ± 0.00 b | 0.00 ± 0.00 b | 0.03 ± 0.02 b | 21.77 ± 9.90 ab | 41.69 ± 31.52 a |
| *Halomonas* | 0.01 ± 0.00 b | 7.20 ± 6.78 b | 6.16 ± 3.72 b | 25.94 ± 4.83 a | 1.77 ± 1.17 b |
| *Bacteroides* | 2.84 ± 3.41 a | 0.68 ± 0.62 ab | 0.30 ± 0.16 b | 1.93 ± 0.86 a | 5.44 ± 8.59 a |
| *Allobaculum* | 7.27 ± 6.40 a | 0.97 ± 1.67 b | 0.10 ± 0.07 b | 1.05 ± 0.41 ab | 0.05 ± 0.10 b |
| *Akkermansia* | 8.17 ± 4.38 a | 0.05 ± 0.07 b | 0.06 ± 0.06 b | 0.86 ± 0.38 ab | 0.00 ± 0.01 b |

| | | | | | |
|---|---|---|---|---|---|
| *Acinetobacter* | 0.00 ± 0.00 b | 0.51 ± 0.61 ab | 0.20 ± 0.18 b | 0.70 ± 0.58 ab | 7.44 ± 9.89 a |
| *Bacillus* | 0.00 ± 0.00 | 2.14 ± 1.90 | 0.73 ± 0.46 | 2.89 ± 1.80 | 0.38 ± 0.32 |
| *Prevotella* | 4.78 ± 4.43 a | 0.09 ± 0.13 b | 0.11 ± 0.15 b | 0.70 ± 0.43 ab | 0.01 ± 0.03 b |
| *Parabacteroides* | 3.78 ± 2.91 a | 0.04 ± 0.04 b | 0.05 ± 0.04 b | 0.85 ± 0.53 ab | 0.00 ± 0.01 b |
| *Staphylococcaceae_Staphylococcus* | 0.00 ± 0.00 b | 0.11 ± 0.13 b | 0.06 ± 0.05 b | 3.96 ± 2.67 a | 0.24 ± 0.15 ab |
| *Achromobacter* | 0.00 ± 0.00 c | 1.28 ± 1.60 ab | 0.55 ± 0.27 b | 1.56 ± 0.26 a | 0.51 ± 0.22 b |
| *Lactobacillus* | 2.45 ± 1.77 a | 0.35 ± 0.53 b | 0.11 ± 0.10 b | 0.57 ± 0.26 ab | 0.11 ± 0.14 b |
| *Prevotellaceae_Prevotella* | 2.83 ± 2.42 a | 0.16 ± 0.11 b | 0.09 ± 0.07 b | 0.46 ± 0.37 | 0.03 ± 0.07 b |

| | | | | | |
|---|---|---|---|---|---|
| | | | | ab | |
| *Pseudomonadaceae_Pseudomonas* | 0.00 ± 0.00 b | 0.28 ± 0.25 a | 0.10 ± 0.08 a | 0.78 ± 0.91 a | 1.47 ± 2.44 a |
| *Serratia* | 0.00 ± 0.00 | 0.54 ± 0.58 | 0.25 ± 0.17 | 1.26 ± 0.87 | 0.53 ± 0.33 |
| *Chelativorans* | 0.00 ± 0.00 b | 0.25 ± 0.31 ab | 0.18 ± 0.10 ab | 0.83 ± 0.24 a | 0.08 ± 0.15 b |
| *Bifidobacterium* | 0.64 ± 0.51 a | 0.06 ± 0.07 b | 0.02 ± 0.04 b | 0.27 ± 0.21 ab | 0.01 ± 0.03 b |
| *Faecalibacterium* | 0.00 ± 0.00 b | 0.25 ± 0.19 a | 0.12 ± 0.09 ab | 0.38 ± 0.28 a | 0.03 ± 0.08 b |
| Other | 1.88 ± 0.80 ab | 1.50 ± 1.39 b | 0.87 ± 0.57 b | 3.49 ± 1.27 a | 1.22 ± 1.77 b |

Data are shown in average ± standard deviation. Different letters indicate the significant difference among different groups for the same index, ANOVA with LSD or Tamhane T2 post-hoc test, $p < 0.05$. N = 6. CTL: control; ATBX: antibiotic; EXC: exercise; KGM: the native KGM; EK: combination of exercise and KGM.

**Table S7 The relative abundance of top 20 taxa in feces at different levels on day 42**

| Day 42 | CTL | ATBX | EXC | KGM | EK |
|---|---|---|---|---|---|
| **Phylum** | | | | | |
| Bacteroidetes | 70.27 ± 27.36 | 77.95 ± 9.21 | 68.01 ± 8.69 | 70.02 ± 20.78 | 78.30 ± 11.06 |
| Firmicutes | 25.32 ± 26.48 | 20.50 ± 9.38 | 11.83 ± 6.13 | 14.31 ± 6.76 | 13.38 ± 3.58 |
| Actinobacteria | 0.40 ± 0.58 b | 0.33 ± 0.35 b | 16.25 ± 10.14 a | 2.50 ± 2.62 ab | 6.54 ± 10.84 ab |
| Proteobacteria | 3.38 ± 6.04 b | 0.89 ± 0.55 b | 3.90 ± 1.11 a | 7.34 ± 10.72 ab | 1.32 ± 0.72 ab |
| Verrucomicrobia | 0.00 ± 0.00 b | 0.32 ± 0.56 ab | 0.00 ± 0.01 b | 5.67 ± 6.86 a | 0.02 ± 0.02 b |
| Tenericutes | 0.45 ± 0.55 a | 0.00 ± 0.01 b | 0.00 ± 0.00 b | 0.00 ± 0.00 b | 0.05 ± 0.05 a |
| Deferribacteres | 0.02 ± 0.03 ab | 0.00 ± 0.00 b | 0.00 ± 0.00 b | 0.00 ± 0.00 b | 0.35 ± 0.50 a |

| | | | | | |
|---|---|---|---|---|---|
| unclassified | 0.06 ± 0.12 | 0.00 ± 0.00 | 0.00 ± 0.00 | 0.00 ± 0.00 | 0.03 ± 0.08 |
| TM7 | 0.08 ± 0.14 a | 0.00 ± 0.00 b | 0.00 ± 0.00 b | 0.00 ± 0.00 b | 0.00 ± 0.00 b |
| Chloroflexi | 0.00 ± 0.00 | 0.00 ± 0.00 | 0.00 ± 0.00 | 0.05 ± 0.12 | 0.00 ± 0.00 |
| Gemmatimonadetes | 0.00 ± 0.00 | 0.00 ± 0.00 | 0.00 ± 0.00 | 0.04 ± 0.10 | 0.00 ± 0.00 |
| Planctomycetes | 0.00 ± 0.00 | 0.00 ± 0.00 | 0.00 ± 0.00 | 0.02 ± 0.06 | 0.00 ± 0.00 |
| Armatimonadetes | 0.00 ± 0.00 | 0.00 ± 0.00 | 0.00 ± 0.00 | 0.02 ± 0.05 | 0.00 ± 0.00 |
| Fusobacteria | 0.00 ± 0.00 | 0.00 ± 0.00 | 0.00 ± 0.00 | 0.01 ± 0.03 | 0.00 ± 0.00 |
| Nitrospirae | 0.00 ± 0.00 | 0.00 ± 0.00 | 0.00 ± 0.00 | 0.01 ± 0.03 | 0.00 ± 0.00 |
| Cyanobacteria | 0.00 ± 0.00 ab | 0.00 ± 0.00 b | 0.00 ± 0.00 b | 0.00 ± 0.00 b | 0.01 ± 0.01 a |
| Acidobacteria | 0.00 ± 0.00 | 0.00 ± 0.00 | 0.00 ± 0.00 | 0.00 ± 0.01 | 0.00 ± 0.00 |
| Crenarchaeota | 0.01 ± 0.01 a | 0.00 ± 0.00 b | 0.00 ± 0.00 b | 0.00 ± 0.00 b | 0.00 ± 0.00 b |
| Euryarchaeota | 0.00 ± 0.00 | 0.00 ± 0.00 | 0.00 ± 0.00 | 0.00 ± 0.00 | 0.00 ± 0.00 |
| WS3 | 0.00 ± 0.00 | 0.00 ± 0.00 | 0.00 ± 0.00 | 0.00 ± 0.00 | 0.00 ± 0.00 |
| Other | 0.00 ± 0.00 | 0.00 ± 0.00 | 0.00 ± 0.00 | 0.00 ± 0.00 | 0.00 ± 0.00 |

| Family | | | | | |
|---|---|---|---|---|---|
| S24_7 | 54.55 ± 21.82 ab | 32.67 ± 11.60 b | 44.26 ± 8.41 ab | 44.44 ± 16.80 ab | 59.28 ± 9.73 a |
| Paraprevotellaceae | 8.46 ± 4.92 a | 25.39 ± 11.92 a | 8.12 ± 4.72 a | 0.00 ± 0.00 b | 8.33 ± 2.03 a |
| Bacteroidaceae | 3.73 ± 2.66 b | 14.23 ± 9.03 a | 7.30 ± 5.52 ab | 18.15 ± 18.33 a | 4.46 ± 2.00 b |
| unclassified | 20.93 ± 26.97 a | 9.81 ± 9.26 a | 1.04 ± 0.69 b | 2.46 ± 3.89 b | 4.08 ± 2.63 ab |
| Porphyromonadaceae | 1.13 ± 0.75 b | 5.56 ± 2.96 ab | 6.27 ± 2.74 a | 5.44 ± 3.49 ab | 3.93 ± 1.52 a |
| Lactobacillaceae | 1.84 ± 1.82 | 3.52 ± 5.58 | 2.24 ± 2.55 | 3.01 ± 2.44 | 5.82 ± 2.69 |
| Bifidobacteriaceae | 0.00 ± 0.00 b | 0.01 ± 0.02 b | 6.41 ± 3.96 a | 2.23 ± 2.48 ab | 5.91 ± 10.73 ab |

| | | | | | |
|---|---|---|---|---|---|
| Erysipelotrichaceae | 0.62 ± 0.38 b | 0.89 ± 1.03 b | 3.29 ± 2.16 a | 4.55 ± 4.22 a | 2.44 ± 1.39 ab |
| Lachnospiraceae | 0.68 ± 0.45 | 4.00 ± 2.91 | 2.51 ± 1.24 | 2.26 ± 2.30 | 1.77 ± 1.25 |
| Coriobacteriaceae | 0.20 ± 0.11 b | 0.31 ± 0.33 b | 9.84 ± 8.54 a | 0.22 ± 0.22 b | 0.63 ± 0.35 ab |
| Ruminococcaceae | 1.28 ± 1.50 | 2.26 ± 1.23 | 2.83 ± 4.65 | 1.11 ± 1.76 | 1.20 ± 0.78 |
| Enterobacteriaceae | 1.24 ± 2.21 ab | 0.05 ± 0.04 b | 0.22 ± 0.47 b | 6.66 ± 10.74 a | 0.31 ± 0.30 ab |
| Verrucomicrobiaceae | 0.00 ± 0.00 b | 0.32 ± 0.56 ab | 0.00 ± 0.01 b | 5.67 ± 6.86 a | 0.02 ± 0.02 b |
| Prevotellaceae | 0.33 ± 0.25 ab | 0.00 ± 0.00 b | 1.56 ± 0.59 a | 1.97 ± 1.95 a | 0.30 ± 0.20 ab |
| Alcaligenaceae | 0.20 ± 0.28 b | 0.17 ± 0.08 b | 2.18 ± 1.05 a | 0.06 ± 0.06 b | 0.38 ± 0.28 ab |
| Rikenellaceae | 1.96 ± 1.40 a | 0.09 ± 0.06 b | 0.49 ± 0.47 ab | 0.00 ± 0.00 bc | 0.00 ± 0.00 c |
| Helicobacteraceae | 0.11 ± 0.08 b | 0.61 ± 0.58 a | 1.18 ± 1.22 a | 0.12 ± 0.17 b | 0.00 ± 0.00 b |
| Turicibacteraceae | 0.00 ± 0.00 b | 0.00 ± 0.00 b | 0.06 ± 0.06 ab | 0.11 ± 0.27 a | 0.00 ± 0.00 b |

| | | | | | |
|---|---|---|---|---|---|
| Pseudomonadaceae | 0.77 ± 1.65 a | 0.00 ± 0.00 b | 0.00 ± 0.00 b | 0.87 ± 0.93 b | 0.00 ± 0.00 b |
| Moraxellaceae | 0.46 ± 1.12 | 0.00 ± 0.00 | 0.00 ± 0.00 | 0.05 ± 0.12 | 0.00 ± 0.01 |
| Other | 1.51 ± 2.92 a | 0.09 ± 0.04 b | 0.22 ± 0.13 ab | 0.62 ± 0.66 a | 1.12 ± 0.62 a |
| **Genus** | | | | | |
| unclassified | 79.72 ± 10.12 a | 45.27 ± 18.28 b | 58.09 ± 11.77 b | 54.39 ± 12.65 b | 65.07 ± 10.22 ab |
| *Prevotella* | 8.46 ± 4.92 a | 25.39 ± 11.92 a | 8.09 ± 4.70 a | 0.00 ± 0.00 b | 7.63 ± 2.19 a |
| *Bacteroides* | 3.73 ± 2.66 b | 14.23 ± 9.03 a | 7.30 ± 5.52 ab | 18.15 ± 18.33 a | 4.46 ± 2.00 b |
| *Parabacteroides* | 1.13 ± 0.75 b | 5.56 ± 2.96 ab | 6.27 ± 2.74 a | 5.44 ± 3.49 ab | 3.93 ± 1.52 a |
| *Lactobacillus* | 1.84 ± 1.82 b | 3.52 ± 5.58 | 2.24 ± 2.55 | 3.01 ± 2.44 ab | 5.82 ± 2.69 a |

| | | | | | |
|---|---|---|---|---|---|
| | | ab | ab | | |
| *Bifidobacterium* | 0.00 ± 0.00 b | 0.01 ± 0.02 b | 6.41 ± 3.96 a | 2.23 ± 2.48 ab | 5.91 ± 10.73 ab |
| *Allobaculum* | 0.45 ± 0.36 | 0.78 ± 1.01 | 2.08 ± 2.21 | 2.91 ± 4.33 | 2.04 ± 1.39 |
| *Akkermansia* | 0.00 ± 0.00 b | 0.32 ± 0.56 ab | 0.00 ± 0.01 b | 5.67 ± 6.86 a | 0.02 ± 0.02 b |
| *Oscillospira* | 0.89 ± 1.30 a | 1.72 ± 1.02 a | 0.95 ± 0.67 a | 0.23 ± 0.23 b | 0.82 ± 0.71 a |
| *Prevotellaceae_Prevotella* | 0.33 ± 0.25 a | 0.00 ± 0.00 b | 1.55 ± 0.59 a | 1.97 ± 1.95 a | 0.30 ± 0.20 ab |
| *Ruminococcaceae_Ruminococcus* | 0.26 ± 0.28 | 0.31 ± 0.24 | 1.70 ± 3.90 | 0.79 ± 1.42 | 0.15 ± 0.08 |
| *Sutterella* | 0.20 ± 0.28 b | 0.16 ± 0.08 b | 2.18 ± 1.05 a | 0.05 ± 0.06 b | 0.36 ± 0.28 ab |
| *Ruminococcus* | 0.06 ± 0.05 | 0.41 ± 0.11 | 0.88 ± 0.88 | 0.94 ± 1.22 | 0.44 ± 0.82 |
| *Coprococcus* | 0.15 ± 0.16 | 0.72 ± 0.93 | 0.17 ± 0.24 | 0.16 ± 0.25 | 0.53 ± 0.44 |
| *Adlercreutzia* | 0.17 ± 0.11 b | 0.14 ± 0.12 b | 0.28 ± 0.12 ab | 0.15 ± 0.22 b | 0.59 ± 0.35 a |

| | | | | | |
|---|---|---|---|---|---|
| *Lachnospiraceae_Clostridium* | 0.01 ± 0.01 b | 0.45 ± 0.62 a | 0.22 ± 0.20 a | 0.30 ± 0.22 a | 0.14 ± 0.20 ab |
| *Erysipelotrichaceae_Clostridium* | 0.01 ± 0.01 b | 0.04 ± 0.05 b | 0.09 ± 0.08 b | 0.83 ± 0.89 a | 0.11 ± 0.14 b |
| *Coprobacillus* | 0.00 ± 0.00 b | 0.00 ± 0.00 b | 0.52 ± 0.96 a | 0.41 ± 0.73 a | 0.02 ± 0.02 ab |
| *Turicibacter* | 0.00 ± 0.00 b | 0.00 ± 0.00 b | 0.06 ± 0.06 ab | 0.87 ± 0.93 a | 0.00 ± 0.00 b |
| *Pseudomonas* | 0.77 ± 1.65 a | 0.00 ± 0.00 b | 0.00 ± 0.00 b | 0.11 ± 0.27 b | 0.00 ± 0.00 b |
| Other | 1.83 ± 3.48 | 0.97 ± 0.71 | 0.91 ± 0.96 | 1.68 ± 1.84 | 1.68 ± 0.84 |

Data are shown in average ± standard deviation. Different letters indicate the significant difference among different groups for the same index, ANOVA with LSD or Tamhane T2 post-hoc test, $p < 0.05$. N = 6. CTL: control; ATBX: antibiotic; EXC: exercise; KGM: the native KGM; EK: combination of exercise and KGM.
